# Hemoglobin Reassembly of Antimicrobial Fragments from the Midgut of *Triatoma infestans*

**DOI:** 10.3390/biom10020261

**Published:** 2020-02-10

**Authors:** Laura Cristina Lima Diniz, Pedro Ismael da Silva Junior

**Affiliations:** 1 Laboratory of Applied Toxinology, Butantan Institute, CEP: 05503-900, São Paulo, SP, Brazil; laura.diniz@butantan.gov.br; 2 Post-Graduation Program Interunits in Biotechnology, USP/IPT/IBU, CEP 05508-900, São Paulo, SP, Brazil

**Keywords:** *Triatoma infestans*, antimicrobial fragments, hemoglobin, triatomines

## Abstract

Hemoglobin is one of the most important molecules of the human body. Beyond its physiological activity, hemoglobins are able to inhibit the growth of several microorganisms. Since 1999, studies have reported that antimicrobial peptides can be produced by blood-feeding insects through hemoglobin digestion, and it has been reported that *Triatoma infestans* can generate an antimicrobial fragment from human fibrinopeptide. Thus *T. infestans* intestinal content was analyzed through Reverse Phase High-Performance Liquid Chromatography (RP-HPLC), the eluted fractions were tested against *Micrococcus luteus*, *Escherichia coli* and *Staphylococcus aureus,* and the active fractions submitted to mass spectrometry. The data obtained were compared to hemoglobin databases to verify the presence of hemoglobin-derived fragments. Ten fractions eluted from chromatography presented antimicrobial activity, and when analyzed through mass spectrometry revealed the presence of 8 murine hemoglobin α-chain fragments and 24 fragments from murine hemoglobin β fragments. Through the compilation of the fragments is possible to obtain over 67% coverage of both sequences. Part of the amino acid sequences corresponds to the sequences already identified on other intestinal contents of arthropods, and are highly conserved between the blood of other wild animals that are the most common intermediate hosts of Chagas’ disease in Brazil and some of the main natural blood source for triatomines.

## 1. Introduction

Hemoglobin is one of the most important molecules of the human body. It is an oxygen/carbon dioxide carrier and has a direct connection with lungs and tissues [1]. Beyond its physiological activity as oxygen carrier, in 1957, Hobson and Hirsch [2] demonstrated that hemoglobins were able to inhibit *Escherichia coli* K12 growth under different ranges of pH and molarity of the buffer system. Even though these alternative biological activities were reported, bioactive peptides isolated from pig hypothalamus were discredited from 1971 to 1980 when it was proven they actually descend from both α or β human hemoglobin chains [3]. These fragments presented activity as corticotrophin, growth hormone and prolactin [4,5,6,7].

Since 1986, several studies demonstrated that fragmentation of hemoglobin can produce series of bioactive peptides. Main reports described opioid activity [8,9,10,11], coronaro-constrictory activity [12] and inhibitory angiotensin converting enzyme activity [13]. Finally, antimicrobial peptides from heme-containing human proteins were identified as components of a larger protein group called hemocidins [14,15].

Although those works described mainly enzymatic, hormonal, analgesic, opioid-like activities and even antimicrobial activity, it was only in 1999 where the first hemoglobin fragment descendent from arthropod digestion was reported with antimicrobial activity against *Micrococcus luteus* [16]. This peptide originated from the digestion of the blood ingested by the tick *Rhicephalus (Boophilus) microplus* and the amino acid content of the peptide ranges from the amino acid residue 33 to the amino acid residue 61 from the bovine hemoglobin alpha-chain. 

Nakajima and his group [17] also demonstrated that other tick, *Ornithodoros moubata*, were able to fragment hemoglobin and generate antimicrobial active fragments on the midgut. The two fragments identified were identical to portions of rabbit hemoglobin. 

Another hemoglobin fragment also originated from this *Rhipicephalus (Boophilus) microplus* midgut content active against several fungi strains, corresponding to 98-114 portion of the bovine hemoglobin alpha-chain, was mostly recent described [18]. 

These studies show that arthropods are able to use their intestinal content for their own protection.

In a recent work, [19] Diniz and her group (2018) demonstrated that the blood-sucking bug, *Triatoma infestans*, is able to absorb human fibrinopeptide A with activity against some bacterial strains, even inside the insect’s hemolymph. 

Therefore, this study was performed in an attempt to elucidate if this same insect is also able to produce antimicrobial peptides during the murine hemoglobin digestion on the midgut. 

## 2. Materials and Methods 

The experiments were performed under the exemption of the *Animal Research Ethics Committee* (CEUAIB – Comitê de ética no uso de animais do Instituto Butantan) nº I–1345/15.

### 2.1. Animals

*Triatoma infestans* were obtained from an axenic culture and kept alive in the vivarium of the Special Laboratory of Toxinology, Butantan Institute (São Paulo, Brazil) at 37 °C and fed every 2 weeks with murine blood.

### 2.2. Bacteria Inoculation and Intestinal Content Collection

One week after blood feeding, adult *T. infestans* were injured with needles soaked in an *Enterobacter cloacae* and *Micrococcus luteus* pool, both at logarithmic-phase growth. After 72 hours, the insect’s midgut was sectioned and the content were scraped and stored at −80 °C until use.

### 2.3. Sample Fractionation

#### 2.3.1. Acid and Solid Phase Extractions

To release the contents of the blood cells, the sample was incubated in acetic acid (2 M) for 30 minutes and centrifuged at 16,000×g for 30 min at 4 °C. The supernatant was injected into coupled Sep-Pack C_18_ cartridges (Waters Associates) equilibrated in 0.1% trifluoroacetic acid (TFA). The sample was eluted in two different acetonitrile (ACN) concentrations (40% and 80%) and then concentrated and reconstituted in ultrapure water.

#### 2.3.2. Reverse Phase High-Performance Liquid Chromatography

Reverse Phase High-Performance Liquid Chromatography (RP-HPLC) separation was performed with a VYDAC semi C_18_ column (Júpiter, 10 × 250 mm) equilibrated with 0.046% TFA. The elution gradient for the 40% ACN fraction was 2% to 60% (v/v) of solution B (0.046% (v/v) TFA in ACN) in solution A (0.046% (v/v) TFA in water) and for the 80% ACN fraction the gradient was 20% to 80% of solution B in solution A. 

RP-HPLC was performed for 120 min at a 1.5 mL/min flow rate. Effluent absorbance was monitored at 225 nm, and the fractions corresponding to absorbance peaks were hand-collected, concentrated under vacuum and reconstituted in ultrapure water.

### 2.4. Liquid Growth Inhibition Assay

A liquid growth inhibition assay was used for evaluating the fractions’ antibacterial activity [20,21]. Lyophilised fractions were suspended in 500 µL Milli-Q water; the assay was carried out using 96-well sterile plates. Twenty µL of the fractions were aliquoted into each well with 80 µL of the bacterial dilution, to 100 µL final volume. Bacteria were cultured in poor nutrient broth (PB) (1.0 g peptone in 100 mL of water containing 86 mM NaCl at pH 7.4; 217 mOsm). Exponential growth phase cultures were diluted to 5 × 10^4^ CFU/mL (DO = 0.001) final concentration [20,22,23]. Sterile water and PB were used as growth control, and streptomycin was used as growth inhibition control. Microtitre plates were incubated for 18 h at 30 °C. Microbial growth was measured by monitoring optical density at 595 nm and assays were performed in triplicate (PerkinElmer Victor 3TM 1420 multilabel counter) [24,25,26].

### 2.5. Mass Spectrometry (LC/MS)

Active antibacterial fractions were analyzed by mass spectrometry Liquid Chromatography – Mass spectrometry (LC-MS/MS) on a LTQ-Orbitrap Velos (Thermo Fisher Scientific, Bremen, Germany) coupled to a liquid nanochromatography system (Easy-nLCII – Thermo Fisher Scientific, Bremen, Germany). The chromatographic step involved using 5 mL of each sample automatically on a C18 pre-column (100 mm I.D. × 50 mm; Jupiter 10 mm, Phenomenex Inc., Torrance, California, United States) coupled to a C18 analytical column (75 mm I.D. × 100 mm; ACQUA 5 mm, Phenomenex Inc.). The eluate was electro-sprayed at 2 kV and 200 °C in positive ion mode. Mass spectra were acquired by a Fourier transform mass analyzers (FTMS); full scan (MS1) involved using 200–2,000 m/z (60,000 resolution at 400 m/z) as mass scan interval with the instrument operated in data dependent acquisition mode, the five most intense ions per scan being selected for fragmentation by collision-induced dissociation. The minimum threshold for selecting an ion for a fragmentation event (MS2) was set to 5000. The dynamic exclusion time used was 15 s, repeating at 30 s intervals.

### 2.6. Computational Analysis and Sequences Alignment

The MS/MS peak list files were submitted to an in-house version of (analyzed through) PEAKS^®^ (Bioinformatics Solutions Inc., Waterloo, Ontario, Canada), screened against hemoglobin databases obtained on Universal Protein (UniProt) [27] and National Center for Biotechnology Information (NCBI) [28] (36,315 sequences and 5866 sequences respectively). Mass spectrometry data were also analyzed on Mascot Deamon® software, version 2.2.2, through MS/MS search using Swissprot database. The results were considered valid only when they were reproducible in a different analysis. Analysis involved 10 ppm error tolerance for precursor ions and 0.6 Da for fragment ions. Metionin oxidation was considered as a variable modification.

The alignment of the primarily peptide sequences were performed with the multiple sequence alignment program Clustal Omega [29] using default parameters. Hemoglobin sequences for comparison were obtained on UniProt using *Monodelphis domestica, Dasypus novemcinctus, Nasua* and *Sus scrofa* as key words. 

The net charge at pH 7 and total charge, possible secondary structure, isoeletric point and total hydrophobic ratio were predicted through the peptide property calculator (copyright © 2015 Innovagen AB) [30], and APD3: Antimicrobial Peptide Calculator and Predictor [31,32].

## 3. Results and Discussion

**Figure 1:**
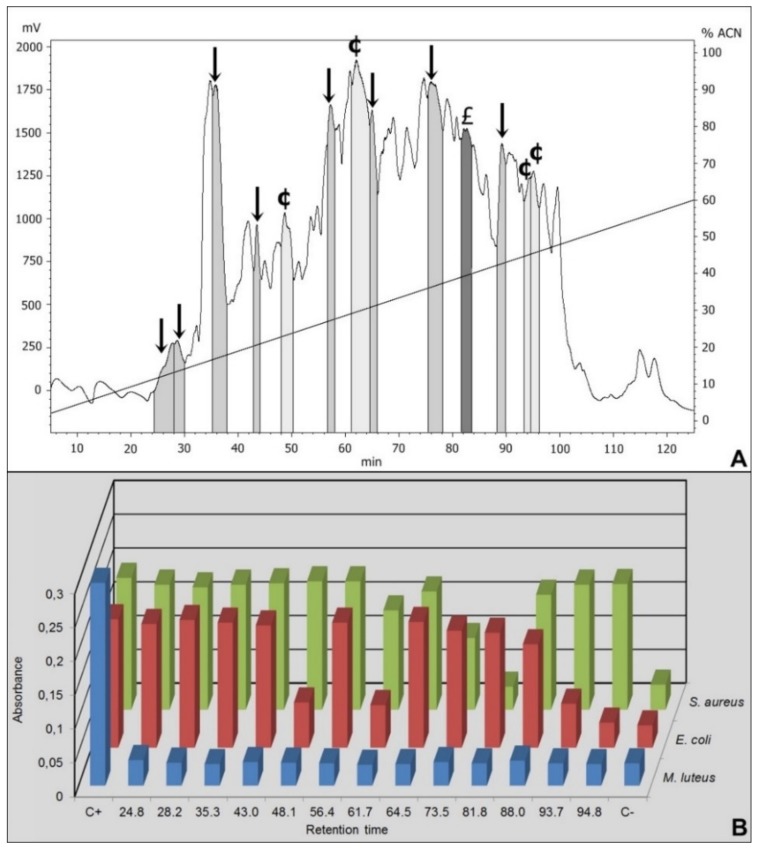
RP-HPLC chromatogram and antimicrobial growth inhibition of 40% ACN Sep-Pack elution samples. (**A**) The fractions isolated from *Triatoma infestans* intestinal content were separated by RP-HPLC using a C_18_ column, eluted with a linear gradient from solution A from 2% to 60% of the solution B run for 120 min. The labeled fractions (**↓**), eluted at 24.8 min, 28.2 min, 35.3 min, 43 min, 56.4 min, 64.5 min, 75.3 min and 88 min, exhibited antimicrobial activity against *Micrococcus luteus*. The labeled fraction (£) eluted at 81.8 min exhibited antimicrobial activity against *Micrococcus luteus* and *Staphylococcus aureus*. The labeled fractions (¢), eluted at 48.1 min, 61.7 min, 93.7 min and 94.8 min, presented antimicrobial activity against *M. luteus* and *Escherichia coli.* (**B**) The absorbance registered after the incubation of the RP-HPLC eluted fractions with *M. luteus, E. coli* and *S. aureus.* C+ is the positive control for bacterial growth, consisting in bacteria incubated only with PB medium. C- is the negative control for bacterial growth, consisting in bacteria incubated with PB medium in the presence of streptomycin.

After the sample preparation, the intestinal content was submitted to an initial fractionation through Sep-Pack C18 (Material and Methods – item 2.3.1), where elutions with 40% and 80% of ACN were performed. The eluted samples were submitted to a second fractionation step in a RP-HPLC (Materials and Methods - item 2.3.2). An antimicrobial screening assay was performed with all the fractions obtained on the second fractionation step (Figure 1A). 

From the 80% ACN elution, 8 fractions presented activity against *M. luteus* but they showed no similarity with any hemoglobin fragments when analyzed through mass spectrometry (data not shown).

We focus only on the 40% ACN fractions. Among the 41 fractions obtained by HPLC from the 40% ACN elution, 37 presented antimicrobial activity against *Micrococcus luteus*. Thus, highlighted on the Figure 1 are only the fractions selected to mass spectrometry. 

Among the selected fractions, eight were active against *M. luteus* (retention time-RT of 24.8 min, 28.2 min, 35.3 min, 43 min, 56.4 min, 64.5 min, 75.3 min and 88 min), the fraction eluted in 81.8 min presented activity against *M. luteus* and *Staphylococcus aureus* and four fractions were active against *M. luteus* and *Escherichia coli* (RT 48.1 min, 61.7 min, 93.7 min and 94.8 min) (Figure 1 and Table 1).

Mass spectrometry data of ten fractions presented similarities to different portions of *Mus musculus* hemoglobin (Supplementary material: Figure S1 to Figure S10), three fractions with eight fragments of α chain (Table 2) and 7 fractions with 24 fragments of the β chain (Table 3).

**Figure 2:**
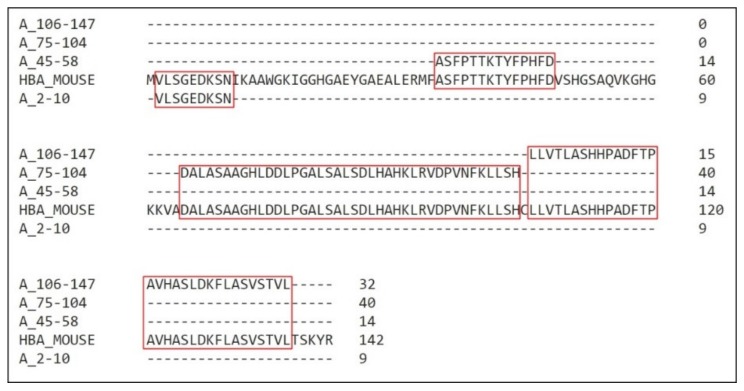
Coverage of *Mus musculus* α hemoglobin sequence. Alignment of the fragments obtained through mass spectrometry with the mouse hemoglobin α chain. Sequence compilations were performed to avoid residues repetitions. The residues coverage is highlighted in red.

**Figure 3:**
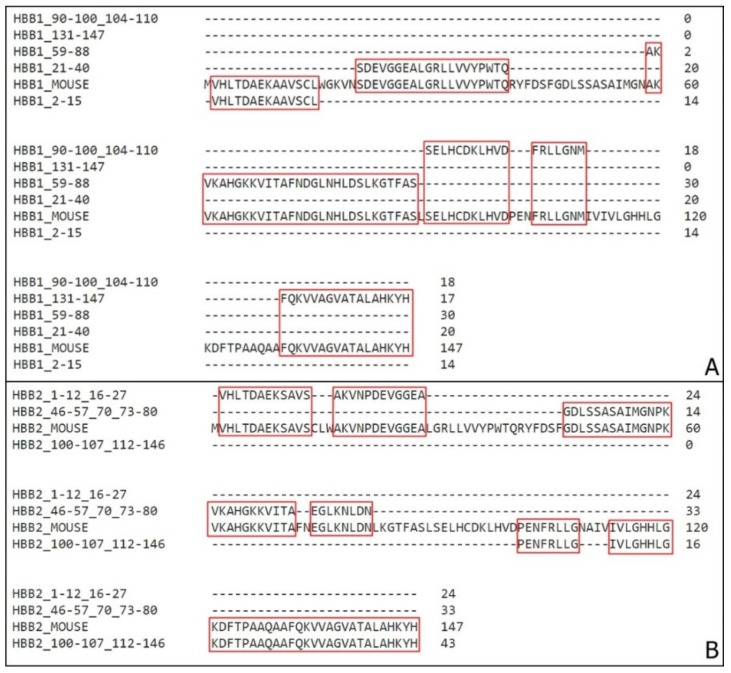
Coverage of *Mus musculus* β hemoglobin sequence. (**A**) Alignment of the fragments obtained through mass spectrometry with the mouse hemoglobin β1 chain (HBB1). (**B**) Alignment of the fragments obtained through mass spectrometry with the mouse hemoglobin β2 chain (HBB2). Sequences compilation was performed to avoid residues repetitions. Highlighted in red are the residues coverage.

Coverage for almost the entire sequence was obtained (Figure 2 and Figure 3). There was approximately 67% coverage for all the sequences.

As demonstrated, more than one sequence was identified on each fraction eluted on the chromatogram. Due to this information, although very unlikely, we cannot exclude the possibility that there could be additional non-Hb peptide or nonpeptide components in each fraction that could contribute to the activity. 

**Figure 4:**
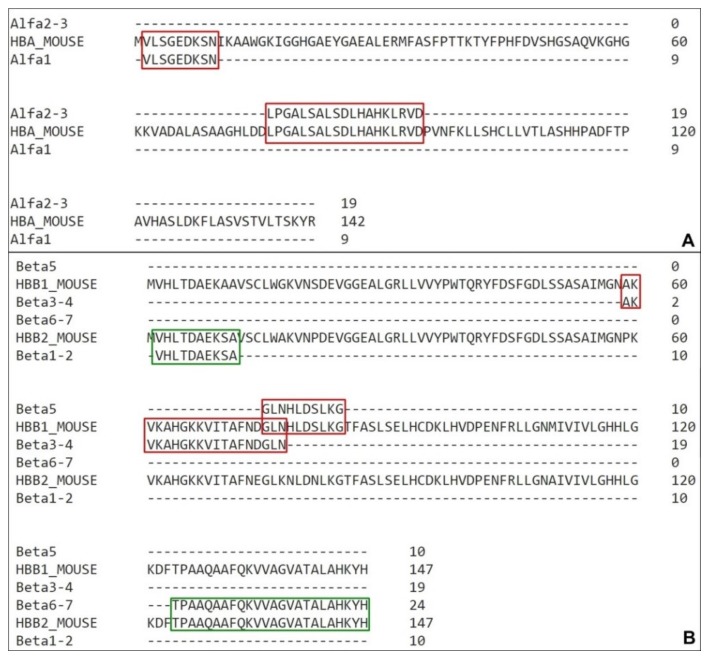
Coverage of *Mus musculus* hemoglobin sequences with high confidence active molecules. (**A**) Alignment of the highest-score fragments obtained through mass spectrometry with the mouse hemoglobin α chain. (**B**) Alignment of the highest score fragments obtained through mass spectrometry with the mouse hemoglobin β1 and β2 chain (HBB1 and HBB2, red and green respectively). Sequences compilation was performed to avoid residues repetitions. The residues coverage is highlighted in red and green.

To determine which sequence may be the responsible for the main antimicrobial activity, other purifications steps would be required. With further analysis, the fragments with higher score were defined (Table 4 and Supplementary Material Figure S11 to Figure S20) and aligned with the complete hemoglobin α and β chain sequences (Figure 4). This result provides more confidence to which fragment present in the sample is the one responsible for the antimicrobial activity. As the aim of the work was to perform a general screening, all the sequences are registered.

Hence this result represents a very important conclusion because it is the first description of a Hemiptera being capable of produce hemoglobin fragments on the intestinal content that can have other biological function for its own benefit, beyond nutrition

These insects have a continuous digestion system. It consists, initially, in the storage of the ingested blood in the anterior midgut (distensible stomach) where it remains undigested, with only lysis of erythrocytes and water absorption take place [33,34]. Then small portions of blood are passed through other digestive and absorptive regions on the mid- and hindgut [35,36].

As previously described by Albritton [37] and Altman and Dittmer [38], with little variation among vertebrate species, whole blood is contained by 80% of water, approximately (corresponding to 94% of the plasma alone). As their mobility is compromised by the volume of blood ingested, and considering that insects do not require this amount of water, its necessary to have a very efficient absorption mechanism and excretion system associated [35].

Along with the water loss, one of the most important process during blood digestion takes place, lysis of erythrocytes also initiates inside the midgut. Red blood cells represent most part of the protein content on the blood, while the plasma alone has a total of 7.41 g of proteins in 100 mL, red blood cells have a total of 36.8 g/100 mL [35].

Due to different pH among insect groups, there are adaptations in the digestive enzymes. 

Hemipterans have acidic midgut contents, with pH decreasing toward the posterior region [39]. The first active degrading molecule in the anterior midgut, as described in *Rhodnius prolixus*, is one haemolisyn small basic peptide [34]. 

Corresponding to the pH alterations through the gut, triatomines have cathepsins as their principal protein degrading enzyme on the posterior midgut [35,40,41,42]. Cathepsins belong to the cysteine protease class and have an optimum activity in pH 5 [43], acting especially in the posterior midgut lumen [44].

After the initial degradation by hemolysins and cathepsins, the generated hemoglobin fragments reach the perimicrovillar spaces where they are cleaved into dipeptides by aminopeptidases and then absorbed [39,45,46].

It has been described that when cleaved, hemoglobin produces several bioactive peptides [8,9,10,11,12].

Antimicrobial peptides (AMPs) are generally small molecules, with under than 50 amino acid residues, and normally have high hydrophobicity and amphipathic features. Cationic charges are one chemical property is the most common AMPs mode of action that can indicate interaction with anionic membranes [47,48,49]. 

The amino acid residue position on the α-helix formation is also essential for membrane interaction, because it allows the peptide to penetrate lipid membranes. This amphipathic characteristic is observed in all the sequences with alpha-helix conformation predicted (Table 5).

**Figure 5:**
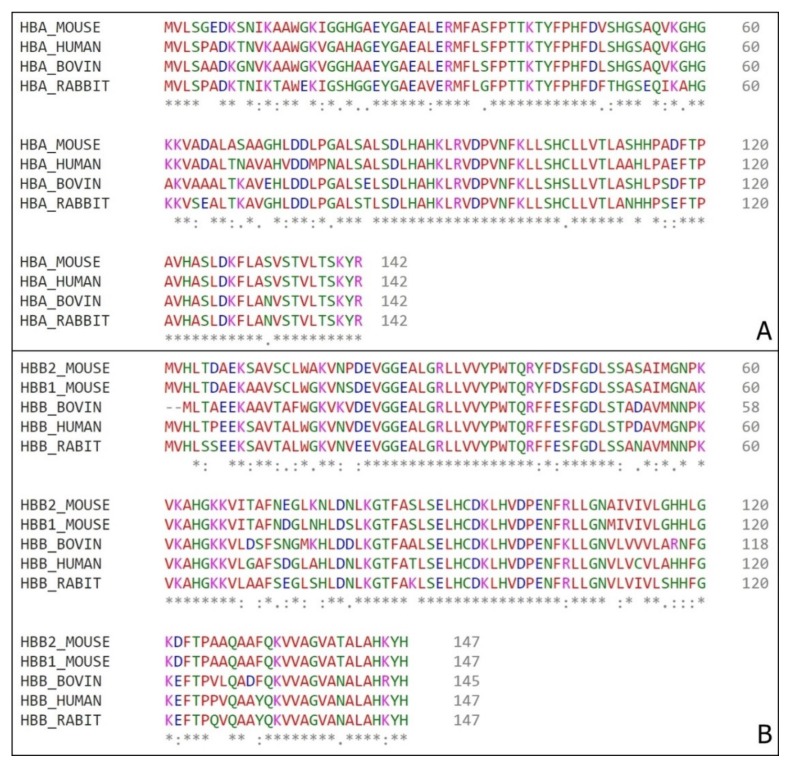
Hemoglobin sequences alignment. (**A**) Alpha chains amino acid comparison from mouse (*Mus musculus)* tag HBA_MOUSE, bovine (*Bos Taurus*) tag HBA_BOVINE, human (*Homo sapiens*) tag HBA_HUMAN and rabbit (*Oryctolagus cuniculus*) tag HBA_RABBIT. (**B**) Beta chains amino acid comparison from mouse (*Mus musculus)* tags HBB1_MOUSE and HBB2_MOUSE for β1 chain and β2 chain respectively, bovine (*Bos Taurus*) tag HBB_BOVINE, human (*Homo sapiens*) tag HBB_HUMAN and rabbit (*Oryctolagus cuniculus*) tag HBB_RABBIT. (*) – position with a single and fully conserved amino acid residue; (:) – position with amino acid residues conserved between groups of strong similar properties; (.) – position with amino acid residues conserved between groups of weakly similar properties.

In 2000, a human hemoglobin fragments with antimicrobial peptides were gathered as a unique family entitled hemocidins [14,15]. Beyond human fragments, bovine and rabbit hemoglobin antimicrobial peptides were also reported [16,17,18]. As these peptides can have different hemoglobin sources, and as this work were developed using mouse, sequence comparison between these species is required (Figure 5). 

The comparison among the hemoglobin chains from different organisms indicate that the sequences are highly conserved, thus there is a probability that the bioactive regions already described in other animals are also highly conserved on the murine protein.

In spite of previous descriptions of antimicrobial fragments from human hemoglobin [48,49,50,51], bovine hemoglobin [52,53,54], comparisons between Cow/Reindeer/Sheep/Pig hemoglobins [54,55,56], we focus on the peptides obtained from in vivo cleavage on insects’ midgut. The described sequences correspond to fractions of alpha chain from cattle and rabbit. They are the α33-61 [16], α98-114 [18] and α1–11/3–19 [17]. 

**Figure 6:**
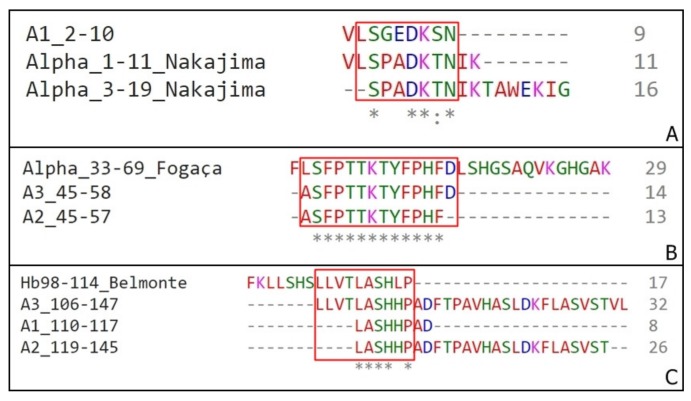
Sequences homology between α chain fragments. (**A**) Alignment of Nakajima’s fragments (Alpha_1–11 and Alpha_3–19) with A1 2–10 isolated on this work. (**B**) Alignment of Fogaça’s fragment (Alpha_33–69) with A2 45–57 and A3 45–58, both isolated on this work. (**C)** Alignment of Belmonte’s fragments (Hb98-114) with A3 106–147, A1 110–117 and A2 119–145 isolated on this work. (*) – position with a single and fully conserved amino acid residue; (:) – position with amino acid residues conserved between groups of strong similar properties.

Interestingly, among the eight α fragments isolated on our work, fraction A1 2–10 is partially homologous to both sequences α1–11 and α3–19 [17], A2 45-57 and A3 45–58 are fully inserted on the α33-61 [16] and Hb98–114 [18] shares approximately five residues with A3 106-147, A1 110–117 and A2 119–145 (Figure 6).

The presence of a smaller fragment inside sequences such as the examples A and B (Figure 6) can indicate the main portion responsible for the peptide antimicrobial activity.

The first important result is the fact that several sequences observed on this work are located on the middle alpha chain sequence, has antimicrobial activity and does not correspond to any of the four sequences identified previously. Examples are A2 α75–95 and A3 α76–104, active against *M. luteus* and *S. aureus*, that are both large sequences and has no amino acid residues overlap. Both A3 106–147 and A2 119–145 are other meaningful examples. Representing a large N-terminal portion of the hemoglobin molecule, it is a fragment that is generated by cathepsins activity on the midgut lumen [46]. This can indicate a different cleavage site between mouse and bovine sequences, or that after the posterior gut digestion, during the aminopeptidases fragmentation, these sequences can form smaller inactive peptides, justifying the lack of identification of these peptides on other studies performed so far.

The Hb98–114 [18] has a specific cytotoxicity against different fungi, the α 1–32/3–32 [17] is active against *S. aureus* and the α 33–61 [16] is active against *M. luteus*. This predilection for Gram-positive bacteria is also observed here. Although the peptides of the murine α-chain identified here haven’t been tested against any fungi strains, all presented some toxicity against *M. luteus* and/or *S. aureus,* and not against *E. coli*. 

A second relevant result it is the description of the production of active beta-chains fragments from murine hemoglobin inside a hematophagous insect midgut. Among the fragments, the sequences inside B4, B6 and B7 HPLC fractions, corresponding to most of Beta1 chain and to the final portion of Beta2 chain are active against *E. coli*. This is an interesting result due to the restriction of Gram-negative activity to fragments from beta chains. 

Due to the fact of the high chains coverage, it was to expect that most part of the fragments here analyzed were compatible with the fragments already described, but our work showed the opposite. The four biggest sequences of α chain presents few or none overlap with the referred before. And as already mentioned it is the first description of an in vivo β chain fragmentation by the insect.

Diniz et al. [19] described a similar event in 2018 where the insect is capable not only to cleavage the protein, but to internalize the fragment produced and use it as a protective factor inside its hemolymph. This fact reinforces the importance of the work, describing the intense bioactive peptide production by the digestion and their importance as a protective factor for the insect against several occasionally pathogens ingested during the blood-feeding. 

**Figure 7:**
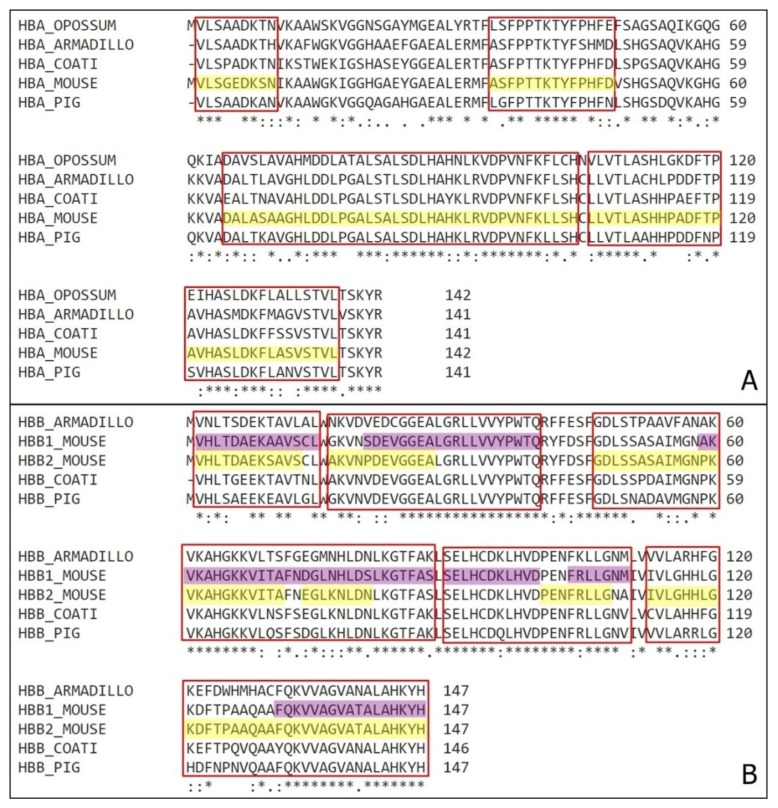
Natural reservoirs organisms’ hemoglobin comparison. (**A**) Comparison of α hemoglobin subunit from *Mus musculus* (HBA_MOUSE) with *Monodelphis domestica* (HBA_OPOSSUM), *Dasypus novemcinctus* (HBA_ARMADILLO), *Nasua nasua* (HBA_COATI) and *Sus scrofa* (HBA_PIG). Highlighted in yellow are the antimicrobial fragments identified on this work, and marked on red are the correspondent sequences on the other animals. (**B**) Comparison of β hemoglobin subunit from *Mus musculus* (HBA_MOUSE) with *Dasypus novemcinctus* (HBA_ARMADILLO), *Nasua nasua* (HBA_COATI) and *Sus scrofa* (HBA_PIG). Highlighted in yellow are the antimicrobial fragments of β1 chain and in purple are the fragments of β2 chain identified on this work, and marked on red are the correspondent sequences on the other animals. (*) – position with a single and fully conserved amino acid residue; (:) – position with amino acid residues conserved between groups of strong similar properties; (.) – position with amino acid residues conserved between groups of weakly similar properties.

Triatomines are insects of socioeconomic importance regarding the role on Chagas disease transmission. The insect contamination occurs when the ingested blood contains trypomastigotes forms of *Trypanossoma cruzi* [57]. Some wild animals are natural reservoirs of the parasite and consequentially are directly connected to the insect contamination with *T. cruzi*. Those natural reservoirs include several species form different taxon and a variety species of triatomines can be infected with *T. cruzi.* Thus, to compare a possible antimicrobial production from other natural reservoir animals, comparisons of hemoglobin sequences were performed (Figure 7). 

Representative organisms from different orders were selected and had their hemoglobin α and β chains compared with *Mus musculus*. The sequences selected belong to the opossum *Monodelphis domestica* (order Marsupialia), the armadillo *Dasypus novemcinctus* (order Xenarthra), the coati *Nasua nasua* (order Carnivora) and the pig *Sus scrofa* (order Artiodactyla). They represent four of the most common intermediate hosts of Chagas’ disease in Brazil and some of the main natural blood source for triatomines. 

All the sequences have high similarity, containing over an average 70% homology, with almost every position with a conserved amino acid or amino acids residues conserved between groups of strong similar properties. 

Considering their highly evolved digestive system, the digestion happens in a very specific way, independent of the species, we can infer that when feeding on other wild animals besides mouse, triatomines (especially *T. infestans*) can also produce the same antimicrobial fragments.

## Figures and Tables

**Table 1:** Antimicrobial results of the HPLC-selected fractions. The highlighted fractions obtained through HPLC with their respectively retention time and antimicrobial activity.

Chromatogram Label	Retention Time	Activity
↓	24.80	*M. luteus*
↓	28.28	*M. luteus*
↓	35.50	*M. luteus*
↓	43.00	*M. luteus*
↓	56.40	*M. luteus*
↓	64.50	*M. luteus*
↓	75.30	*M. luteus*
↓	88.80	*M. luteus*
**¢**	48.10	*M. luteus and E. coli*
**¢**	61.70	*M. luteus and E. coli*
**¢**	93.70	*M. luteus and E. coli*
**¢**	94.8	*M. luteus and E. coli*
£	81.80	*M. luteus and S. aureus*

**Table 2:** Mass spectrometry data database search (α chain results). Fraction A1 (eluted at 24.8 min) has two peptides corresponding to the fragments 2–10 and 110–117 of the hemoglobin α chain. Fraction A2 (eluted at 64.5 min) has three peptides corresponding to the fragments 45–57, 75–95 and 119–145 of the hemoglobin α chain. Fraction A3 (eluted at 81.8 min) has three peptides corresponding to the fragments 45–58, 76–104 and 106–147 of the hemoglobin α chain.

Fraction	Retention Time	Fragment
A1	24.80	VLSGEDKSN (α 2-10)
A1	24.80	LASHHPAD (α 110-117)
A2	64.50	ASFPTTKTYFPHF (α 45-57)
A2	64.50	DALASAAGHLDDLPGALSALSDLHAHKLRVD (α 75–95)
A2	64.50	LASHHPADFTPAVHASLDKFLASVST (α 119–145)
A3	81.80	ASFPTTKTYFPHFD (α 45–58)
A3	81.80	ALASAAGHLDDLPGALSALSDLHAHKLRVDPVNFKLLSH (α 76–104)
A3	81.80	LLVTLASHHPADFTPAVHASLDKFLASVSTVL (α 106–147)

**Table 3:** Mass spectrometry data database search (β chain results). Fraction B1 (eluted at 28.28 min) has two peptides corresponding to the fragments 2–10 and 56–77 of the hemoglobin β chain. Fraction B2 (eluted at 35.5 min) has five peptides corresponding to the fragments 1-10, 16–27, 49–70, 73–80 and 122–136 of the hemoglobin β chain. Fraction B3 (eluted at 43 min) has three peptides corresponding to the fragments 1–12, 58–72 and 112–129 of the hemoglobin β chain. Fraction B4 (eluted at 48.1 min) has three peptides corresponding to the fragments 2–14, 59–88 and 135–147 of the hemoglobin β chain. Fraction B5 (eluted at 56.4 min) has four peptides corresponding to the fragments 34–40, 59–71, 75–86 and 90–100 of the hemoglobin β chain. Fraction B6 (eluted at 61.7 min) has five peptides corresponding to the fragments 2–15, 21–33, 90–100, 104–110 and 131–147 of the hemoglobin β chain. Fraction B7 (eluted at 93.7 min) has two peptides corresponding to the fragments 100–107 and 112–146 of the hemoglobin β chain.

Fraction	Retention Time	Sequence
B1	28.28	HLTDAEKSA (β2_ 2-10)
B1	28.28	GDLSSASAIMGN (β2_ 46-57)
B2	35.50	VHLTDAEKSA (β2_1-10)
B2	35.50	AKVNPDEVGGEA (β2_16-27)
B2	35.50	SSASAIMGNPKVKAHGKKVITA (β2_ 49-70)
B2	35.50	EGLKNLDN (β2_73-80)
B2	35.50	FTPAAQAAFQKVVAG (β2_122-136)
B3	43.00	VHLTDAEKSAVS (β2_1-12)
B3	43.00	PKVKAHGKKVITAFN (β2_58-72)
B3	43.00	IVLGHHLGKDFTPAAQAA (β2_112-129)
B4	48.10	VHLTDAEKAAVSC (β1_ 2-14)
B4	48.10	AKVKAHGKKVITAFNDGLNHLDSLKGTFAS (β1_ 59-88)
B4	48.10	AGVATALAHKYH (β1_135-147)
B5	56.40	VVYPWTQ (β1_34-40)
B5	56.40	AKVKAHGKKVITA (β1_ 59-71)
B5	56.40	GLNHLDSLKGTF (β1_75-86)
B5	56.40	SELHCDKLHVD (β1_90-100)
B6	61.70	VHLTDAEKAAVSCL (β1_2-15)
B6	61.70	SDEVGGEALGRLL (β1_21-33)
B6	61.70	SELHCDKLHVD (β1_ 90-100)
B6	61.70	FRLLGNM β (β1_104-110)
B6	61.70	FQKVVAGVATALAHKYH (β1_131-147)
B7	93.70	PENFRLLG (β2_100-107)
B7	93.70	IVLGHHLGKDFTPAAQAAFQKVVAGVATALAHKYH (β2_112-146)

**Table 4:** Sequences with highest score. On the hemoglobin α chain, three fragments are the most confident sequence. Fraction A1 (eluted at 24.8 min) has the fragment from the amino acid 2 to 10, Fraction A2 (eluted at 64.5 min) and Fraction A3 (eluted at 81.8 min) have the fragment from the amino acid 77 to 95. On the hemoglobin β chain, the most confident sequence on the Fraction B1 (eluted at 28.28 min) is the fragments from the amino acid 2 to 10, on the Fraction B2 (eluted at 35.5 min) is the fragment from the amino acid 1 to 10, on the Fraction B3 (eluted at 43 min) is the fragments 1–12, 58–72 and 112–129 of the hemoglobin β chain. Fraction B4 (eluted at 48.1 min) has three peptides corresponding to the fragments 2–14, 59–88 and 135–147 of the hemoglobin β chain. Fraction B5 (eluted at 56.4min) has four peptides corresponding to the fragments 34–40, 59–71, 75–86 and 90–100 of the hemoglobin β chain. Fraction B6 (eluted at 61.7 min) has five peptides corresponding to the fragments 2–15, 21–33, 90–100, 104–110 and 131–147 of the hemoglobin β chain. Fraction B7 (eluted at 93.7 min) has two peptides corresponding to the fragments 100–107 and 112–146 of the hemoglobin β chain.

Fraction	Retention Time	Fragment
A1	24.80	VLSGEDKSN (α 2–10)
A2	64.50	LPGALSALSDLHAHKLRVD (α 77–95)
A3	81.80	LPGALSALSDLHAHKLRVD (α 77–95)
B1	28.28	HLTDAEKSA (β2_ 2–10)
B2	35.50	VHLTDAEKSA (β2_1–10)
B3	43.00	AKVKAHGKKVITAFND (β1_59–74)
B4	48.10	AKVKAHGKKVITAFNDGLN (β1_ 59–77)
B5	56.40	GLNHLDSLKG (β1_75–84)
B6	61.70	FQKVVAGVATALAHKYH (β1_131–147)
B7	93.70	TPAAQAAFQKVVAGVATALAHKYH (β2_123–147)

**Table 5:** Fragments chemical properties. Chemical features of the fragments with alpha-helix predicted structure. H.R. stands for hydrophobic residue. α stands for alpha chain, β1 stands for beta-1 chain and β2 stands for beta-2 chain.

Fraction	Fragment	Charge pH 7	Total Hydrophobic Ratio	Same Surface brk H.R.*
A2	α 75-95	-2.7	48%	12
A2	α 119-145	-0.7	46%	8
A3	α 76-104	-0.6	48%	14
A3	α 106-147	-0.7	53%	14
B1	β2_2-10	-0.9	33%	3
B1	β2_46-57	-1	41%	4
B2	β2_1-10	-0.9	40%	3
B2	β2_49-70	4.1	40%	6
B2	β2_73-80	-1	25%	2
B2	β2_122-136	1	60%	6
B3	β2_1-12	-0.9	41%	3
B3	β2_58-72	4.1	40%	4
B3	β2_112-129	0.2	50%	6
B4	β1_2-14	-1	53%	4
B4	β1_59-88	3.2	40%	8
B4	β1_135-147	1.2	50%	4
B5	β1_59-71	4.1	46%	3
B5	β1_75-86	0.1	33%	4
B6	β1_2-15	-1	57%	6
B6	β1_21-33	-2	38%	3
B6	β1_104-110	1	57%	3
B6	β1_131-147	2.2	52%	6
B7	β2_100-107	0	37%	2
B7	β2_112-146	2.4	51%	14

## Supplementary Materials

The following are available online at https://www.mdpi.com/2218-273X/10/2/261/s1, Figure S1: Deconvolution of two peptides from the fraction A1 (eluted at 24.8 min) corresponding to the fragments 2–10 and 110–117 of the hemoglobin α chain, Figure S2: Deconvolution of three peptides from the fraction A2 (eluted at 64.5 min) corresponding to the fragments 45–57, 75–95 and 119–145 of the hemoglobin α chain, Figure S3: Deconvolution of three peptides from the fraction A3 (eluted at 81.8 min) corresponding to the fragments 45–58, 76–104 and 106–147 of the hemoglobin α chain, Figure S4: Deconvolution of two peptides from the fraction B1 (eluted at 28.28 min) corresponding to the fragments 2–10 and 56–77 of the hemoglobin β chain, Figure S5. Deconvolution of five peptides from the fraction B2 (eluted at 28.28 min) corresponding to the fragments 1–10, 16–27, 49–70, 73–80 and 122–136 of the hemoglobin β chain, Figure S6. Deconvolution of three peptides from the fraction B3 (eluted at 43 min) corresponding to the fragments 1–12, 58–72 and 112–129 of the hemoglobin β chain, Figure S7. Deconvolution of three peptides from the fraction B4 (eluted at 48.1 min) corresponding to the fragments 2–14, 59–88 and 135–147 of the hemoglobin β chain, Figure S8. Deconvolution of four peptides from the fraction B5 (eluted at 56.4 min) corresponding to the fragments 34–40, 59–71, 75–86 and 90–100 of the hemoglobin β chain, Figure S9. Deconvolution of five peptides from the fraction B6 (eluted at 61.7 min) corresponding to the fragments 2–15, 21–33, 90–100, 104–110 and 131–147 of the hemoglobin β chain, Figure S10: Deconvolution of two peptides from the fraction B2 (eluted at 93.7 min) corresponding to the fragments 100–107 and 112–146 of the hemoglobin β chain, Figure S11: Deconvolution of the main peptide from the fraction A1 (eluted at 24.8 min) corresponding to the fragment 2–10 of the hemoglobin α chain, Figure S12: Deconvolution of the main peptide from the fraction A2 (eluted at 64.5 min) corresponding to the fragment 77–95 of the hemoglobin α chain, Figure S13: Deconvolution of the main peptide from the fraction A3 (eluted at 81.8 min) corresponding to the fragment 77–95 of the hemoglobin α chain, Figure S14: Deconvolution of the main peptide from the fraction B1 (eluted at 28.8 min) corresponding to the fragment 2–10 of the hemoglobin β2 chain, Figure S15: Deconvolution of the main peptide from the fraction B2 (eluted at 35.5 min) corresponding to the fragment 1-10 of the hemoglobin β2 chain, Figure S16: Deconvolution of the main peptide from the fraction B3 (eluted at 43.0 min) corresponding to the fragment 59–74 of the hemoglobin β1 chain, Figure S17: Deconvolution of the main peptide from the fraction B4 (eluted at 48.1 min) corresponding to the fragment 59–77 of the hemoglobin β1 chain, Figure S18: Deconvolution of the main peptide from the fraction B5 (eluted at 56.4 min) corresponding to the fragment 75–84 of the hemoglobin β1 chain, Figure S19: Deconvolution of the main peptide from the fraction B6 (eluted at 61.7 min) corresponding to the fragment 131–147 of the hemoglobin β1 chain, Figure S20: Deconvolution of the main peptide from the fraction B7 (eluted at 93.7 min) corresponding to the fragment 123–147 of the hemoglobin β2 chain.
