# Supplementary material for: Hemoglobin Reassembly of Antimicrobial Fragments from the Midgut of Triatoma infestans
Source: Biomolecules. 2020 Feb 10;10(2):261. doi: 10.3390/biom10020261 (PMC7072205; doi:10.3390/biom10020261)

Supplementary Material

Figure S1: Deconvolution of two peptides from the fraction A1 (eluted at 24.8min) corresponding to the fragments 2-10 and 110-117 of hemoglobin α chain.

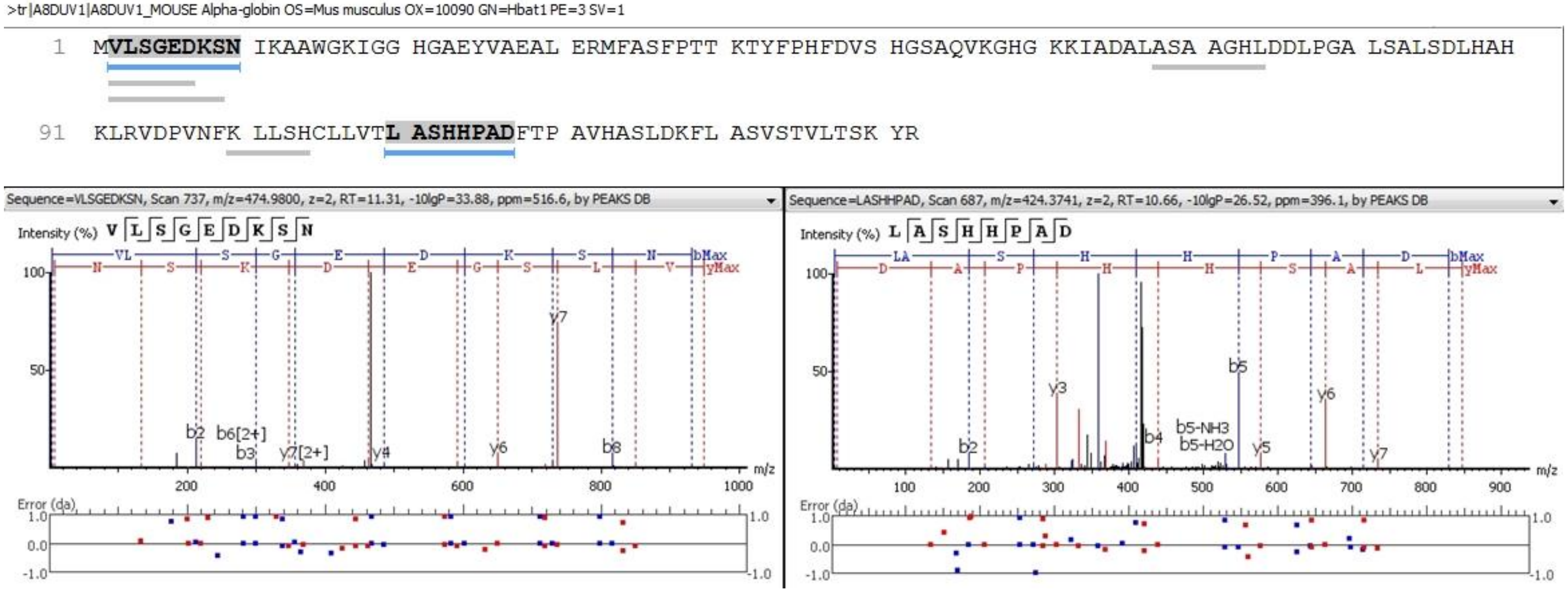

Figure S2: Deconvolution of three peptides from the fraction A2 (eluted at 64.5min) corresponding to the fragments 45-57, 75-95 and 119-145 of hemoglobin α chain.

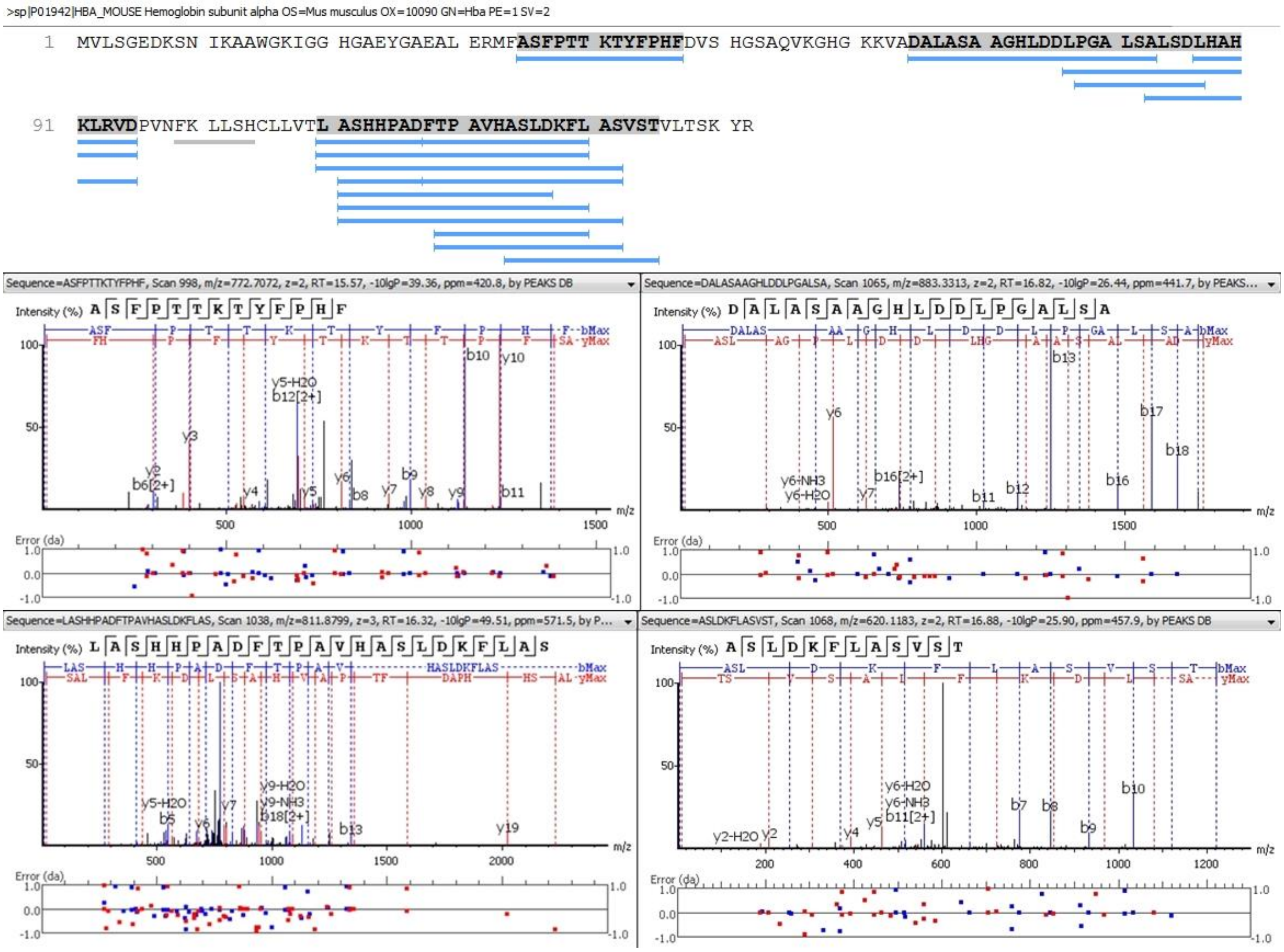

Figure S3: Deconvolution of three peptides from the fraction A3 (eluted at 81.8min) corresponding to the fragments 45-58, 76-104 and 106-147 of hemoglobin α chain

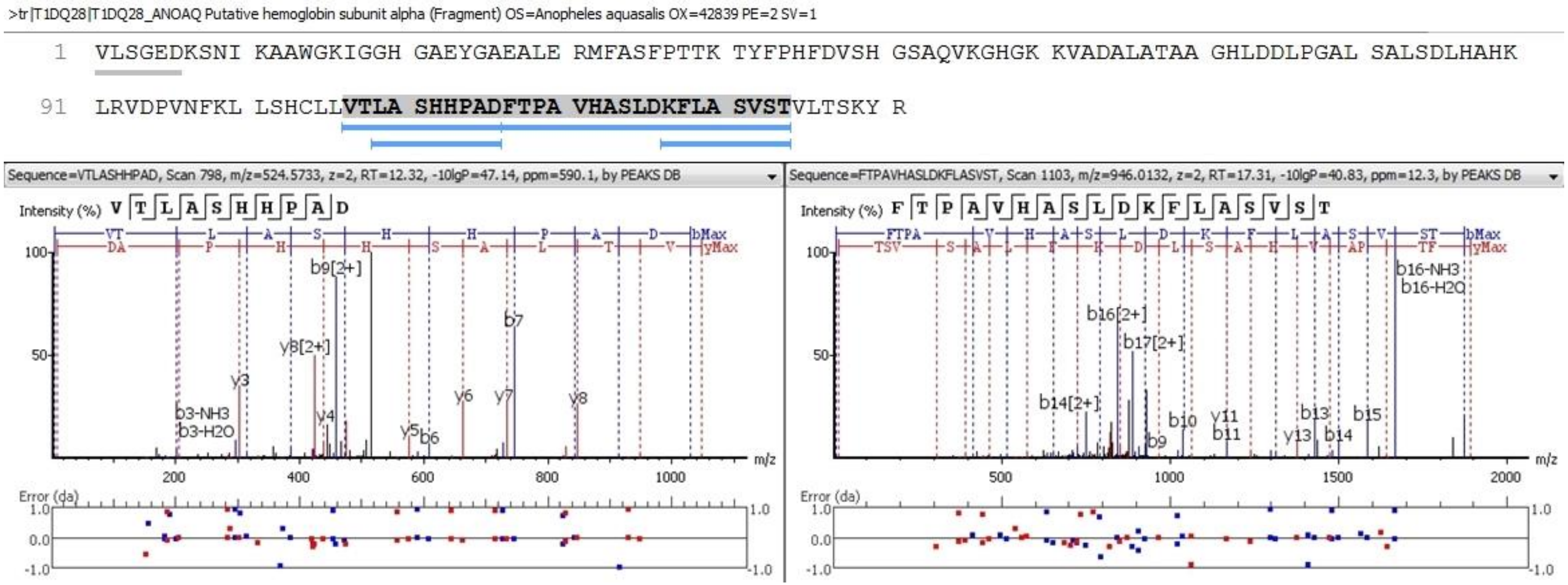

Figure S4: Deconvolution of two peptides from the fraction B1 (eluted at 28.28min) corresponding to the fragments 2-10 and 56-77 of hemoglobin β chain.

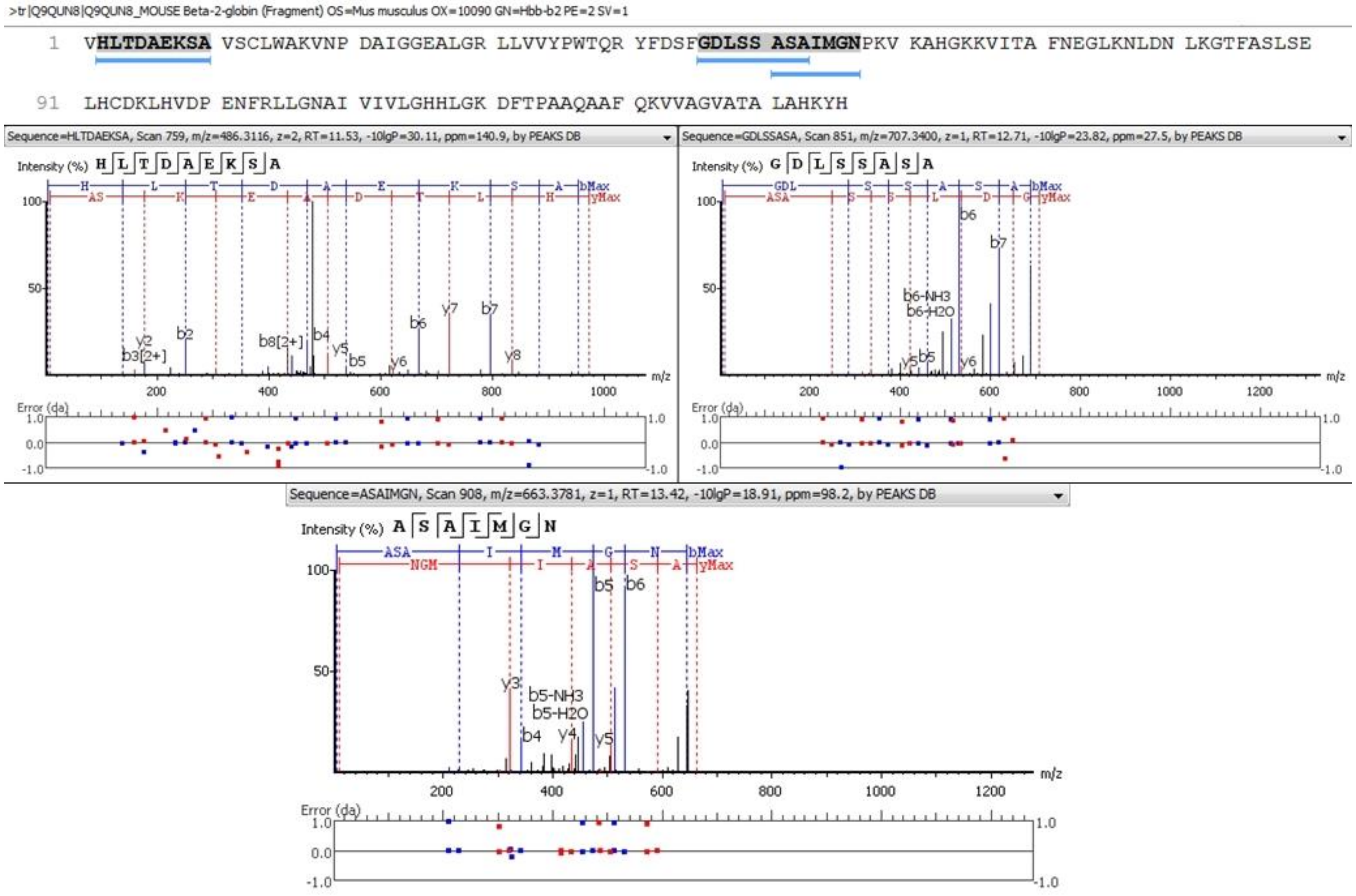

Figure S5: Deconvolution of five peptides from the fraction B2 (eluted at 28.28min) corresponding to the fragments 1-10, 16-27, 49-70, 73-80 and 122-136 of hemoglobin β chain.

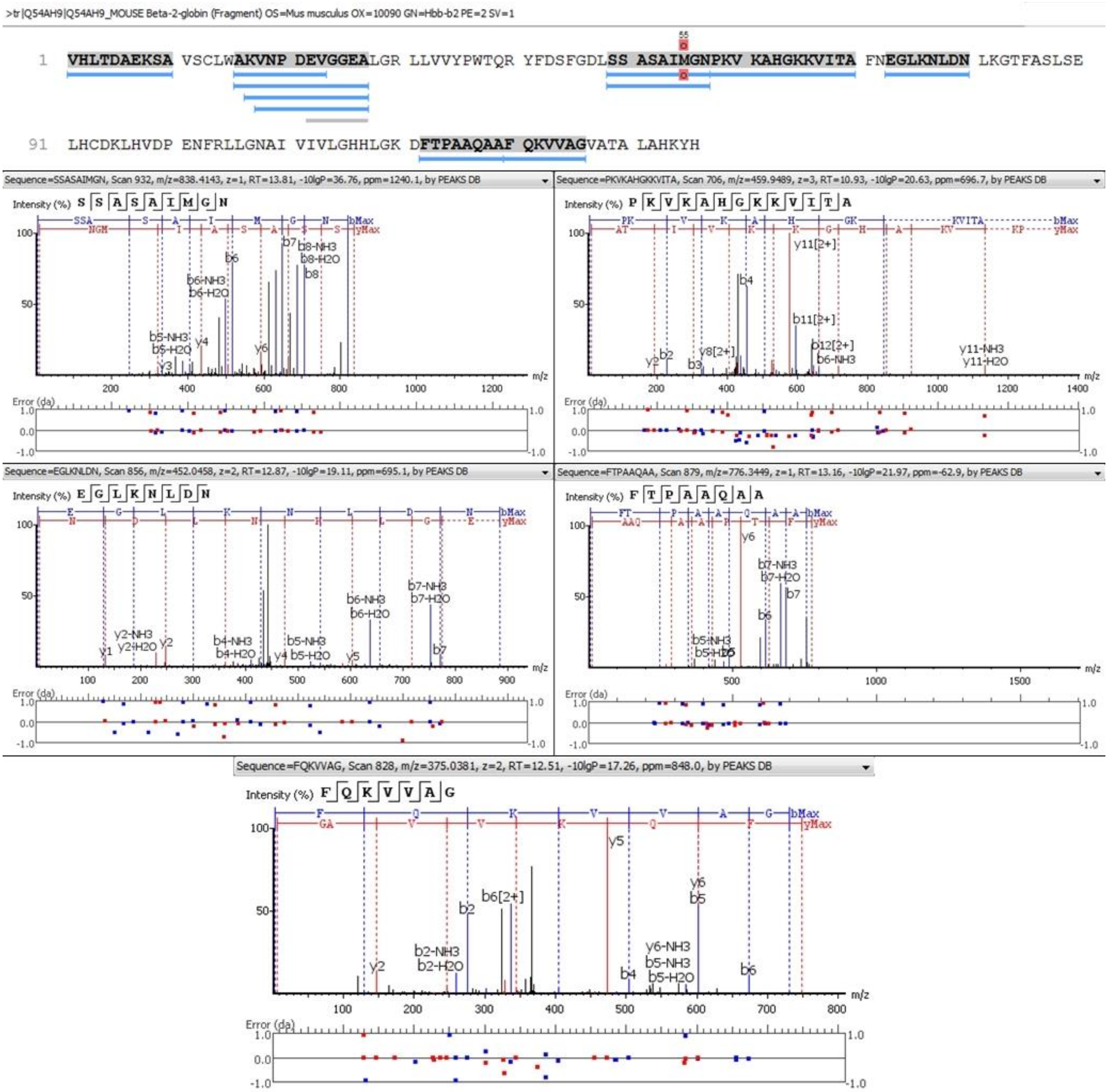

Figure S6: Deconvolution of three peptides from the fraction B3 (eluted at 43min) corresponding to the fragments 1-12, 58-72 and 112-129 of hemoglobin β chain.

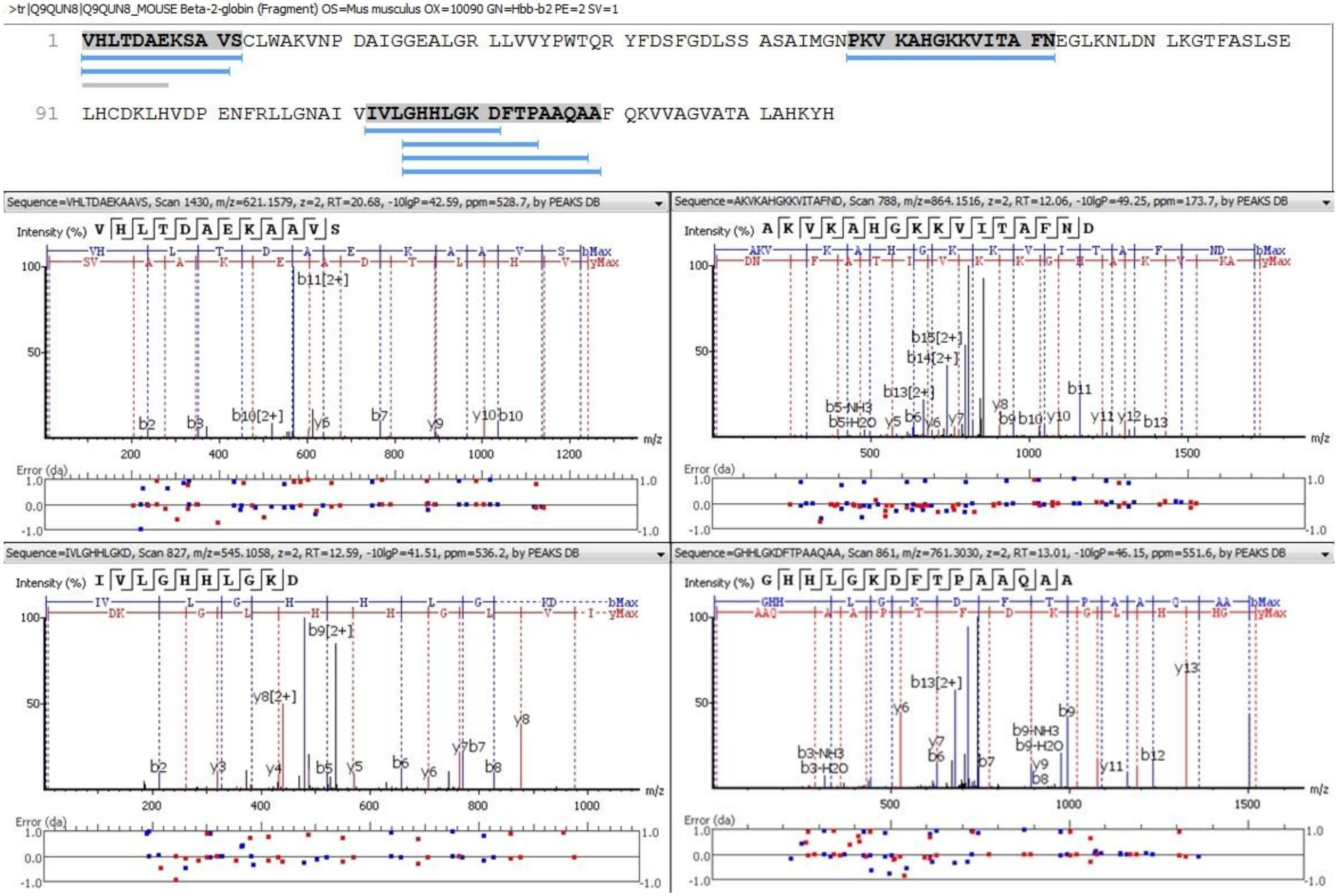

Figure S7: Deconvolution of three peptides from the fraction B4 (eluted at 48.1min) corresponding to the fragments 2-14, 59-88 and 135-147 of hemoglobin β chain.

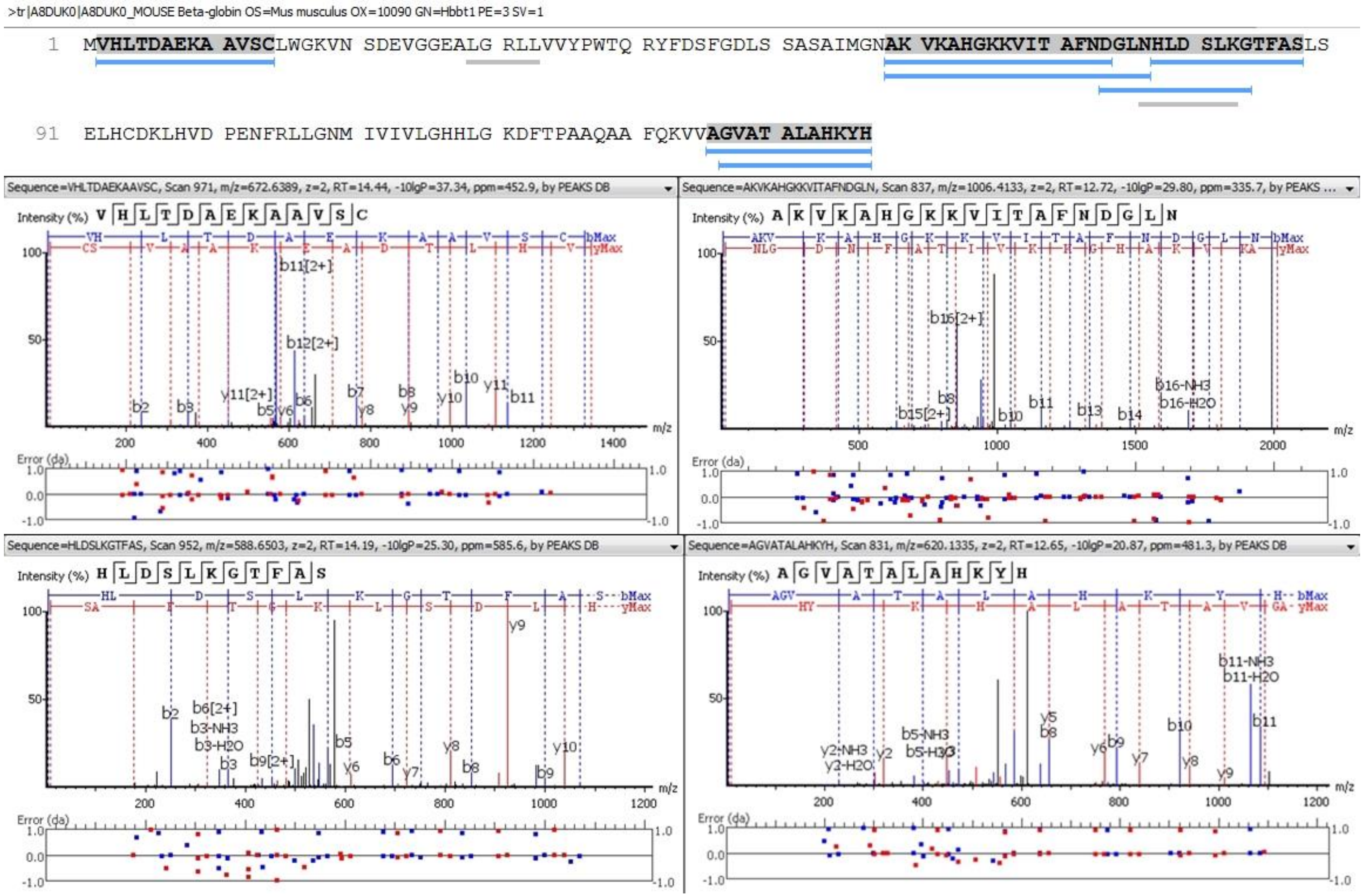

Figure S8: Deconvolution of four peptides from the fraction B5 (eluted at 56.4min) corresponding to the fragments 34-40, 59-71, 75-86 and 90-100 of hemoglobin β chain.

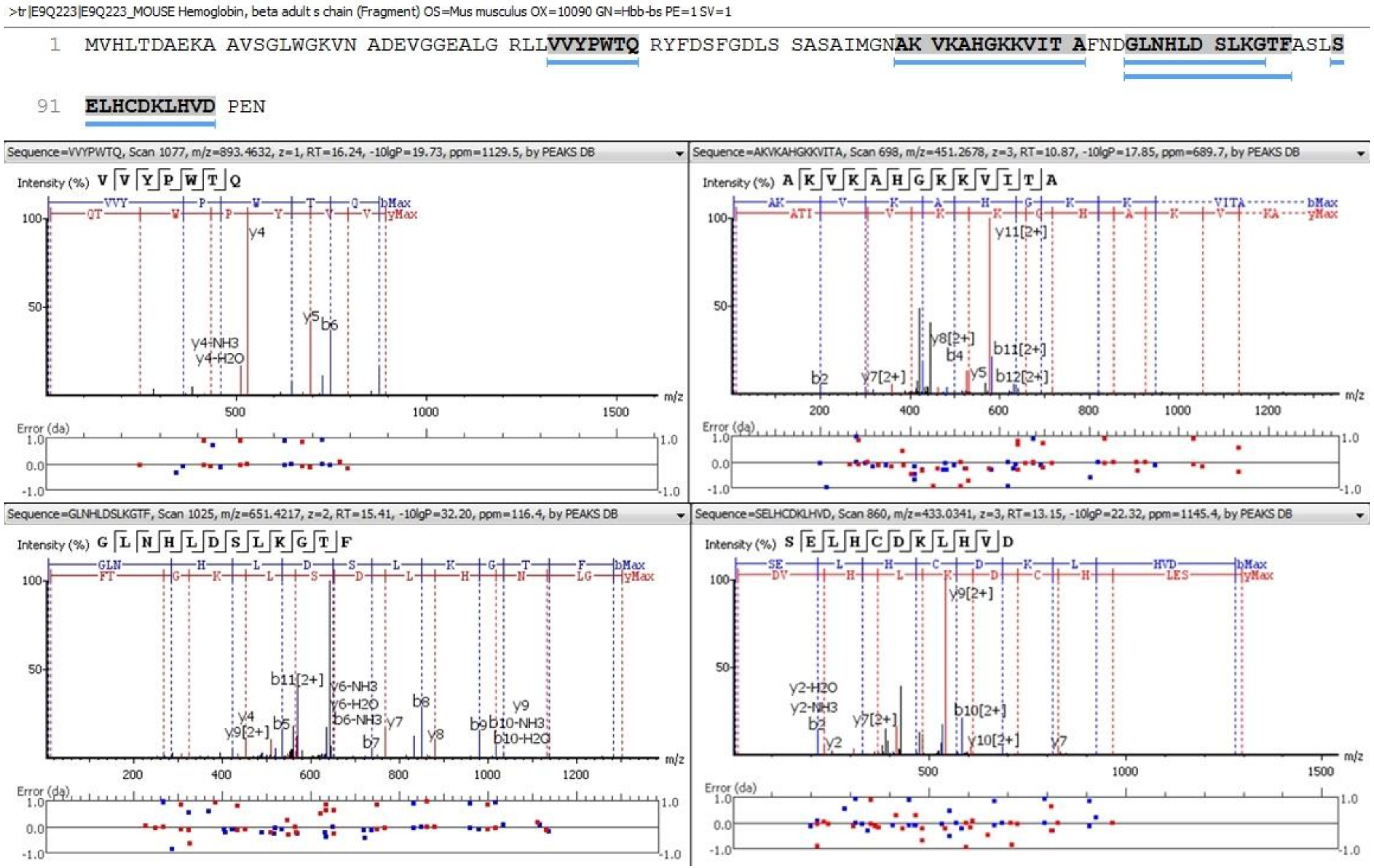

Figure S9: Deconvolution of five peptides from the fraction B6 (eluted at 61.7min) corresponding to the fragments 2-15, 21-33, 90-100, 104-110 and 131-147 of hemoglobin β chain.

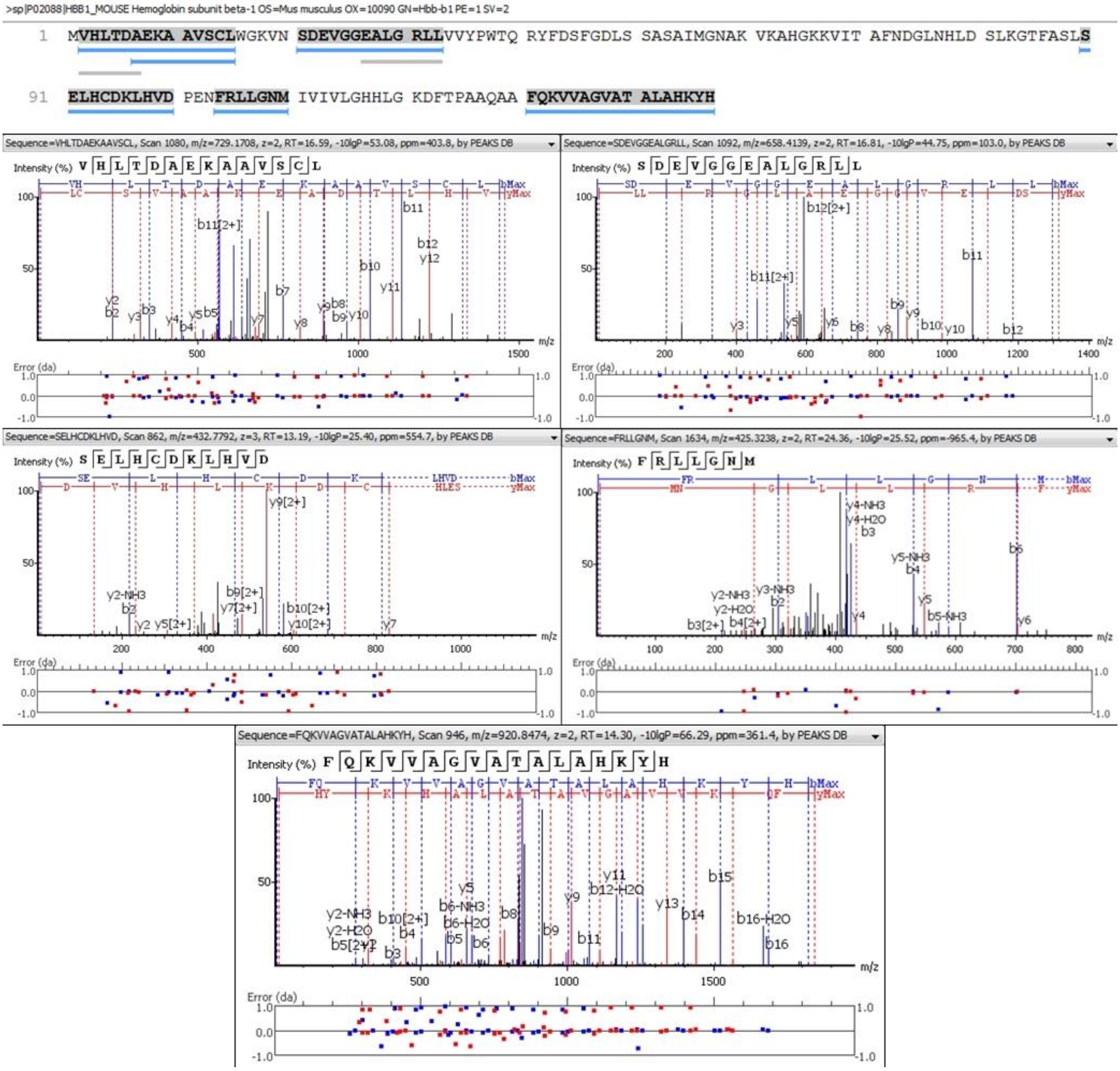

Figure S10: Deconvolution of two peptides from the fraction B2 (eluted at 93.7min) corresponding to the fragments 100-107 and 112-146 of hemoglobin β chain.

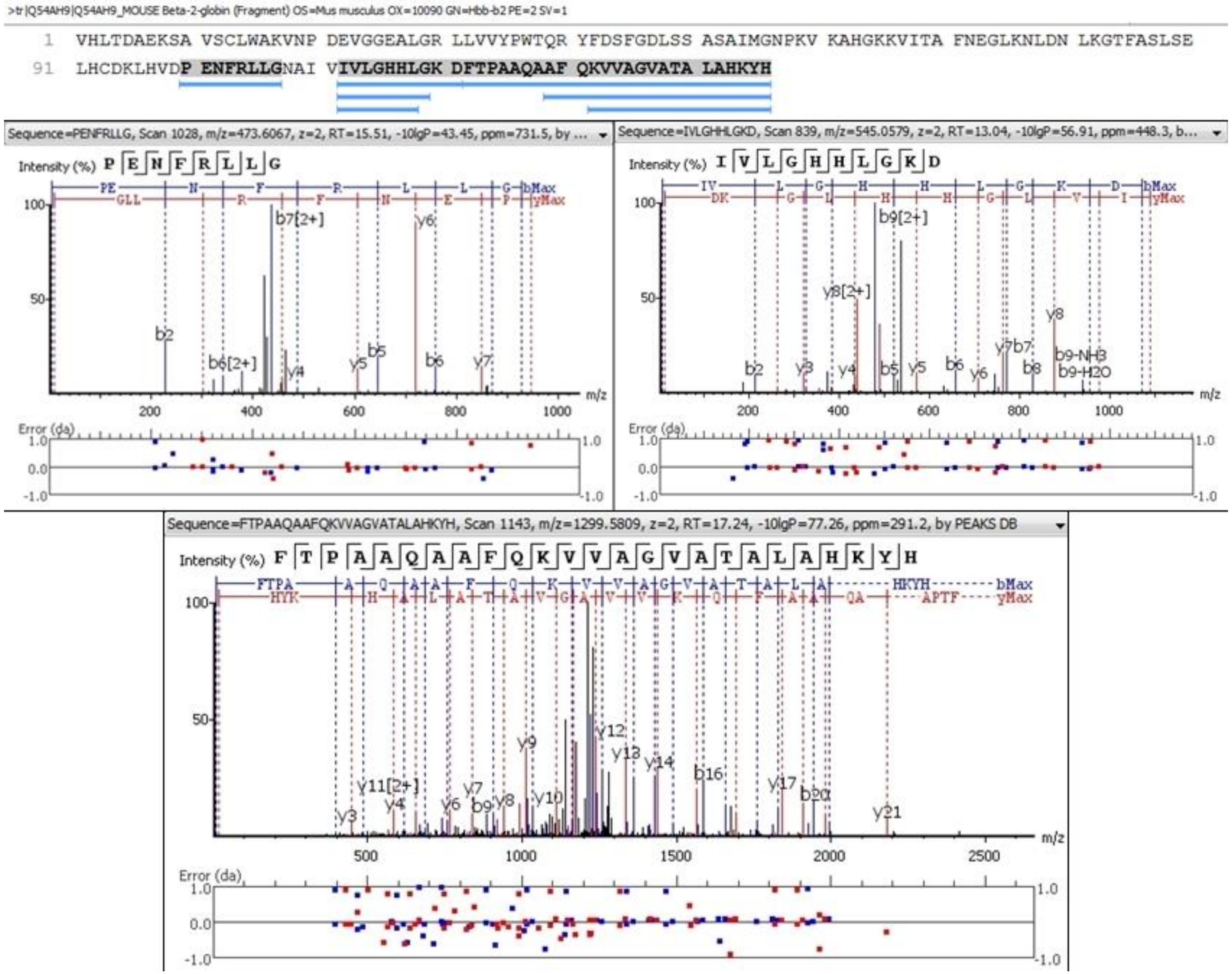

Figure S11: Deconvolution of the main peptide from the fraction A1 (eluted at 24.8min) corresponding to the fragment 2-10 of hemoglobin α chain.

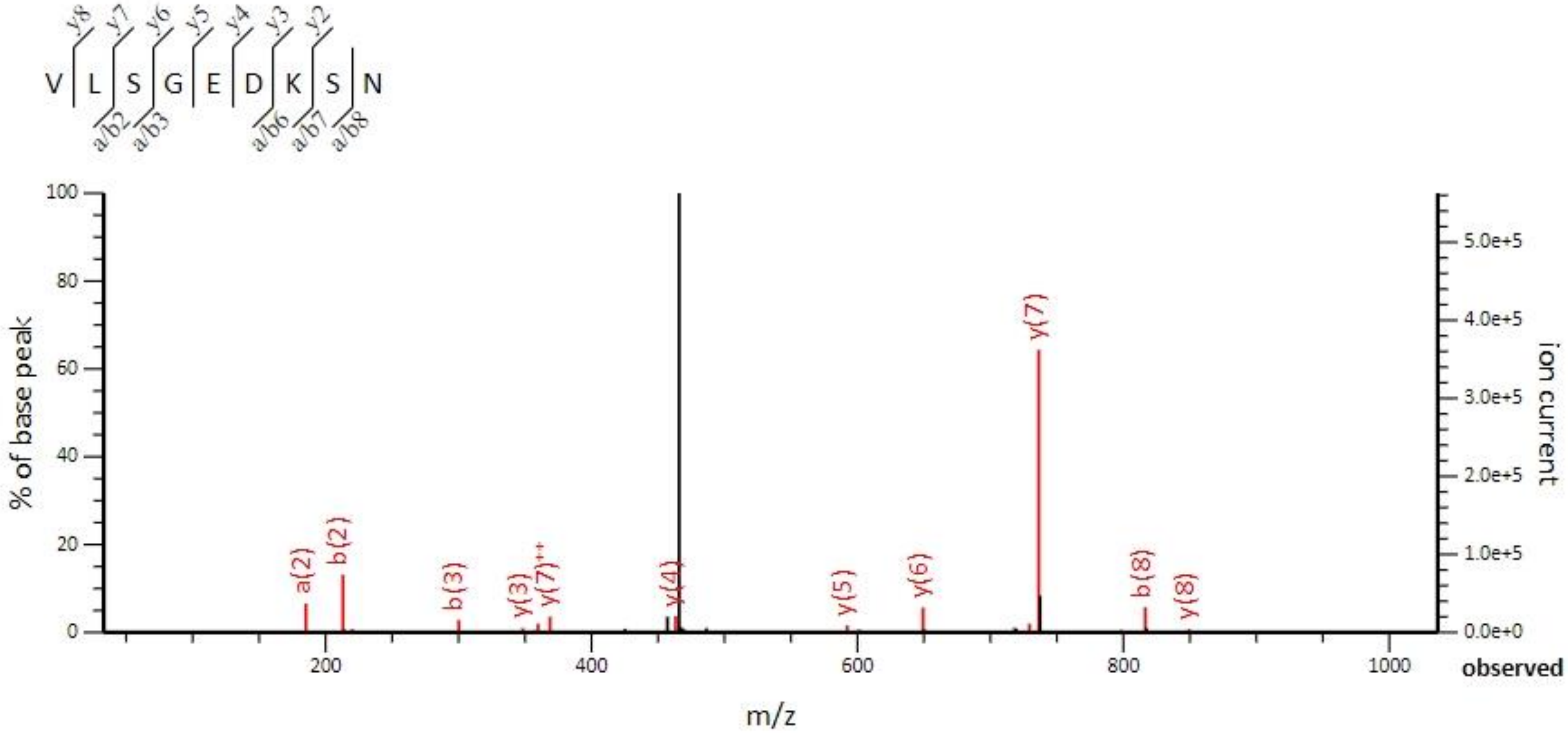

Figure S12: Deconvolution of the main peptide from the fraction A2 (eluted at 64.5 min) corresponding to the fragment 77-95 of hemoglobin α chain.

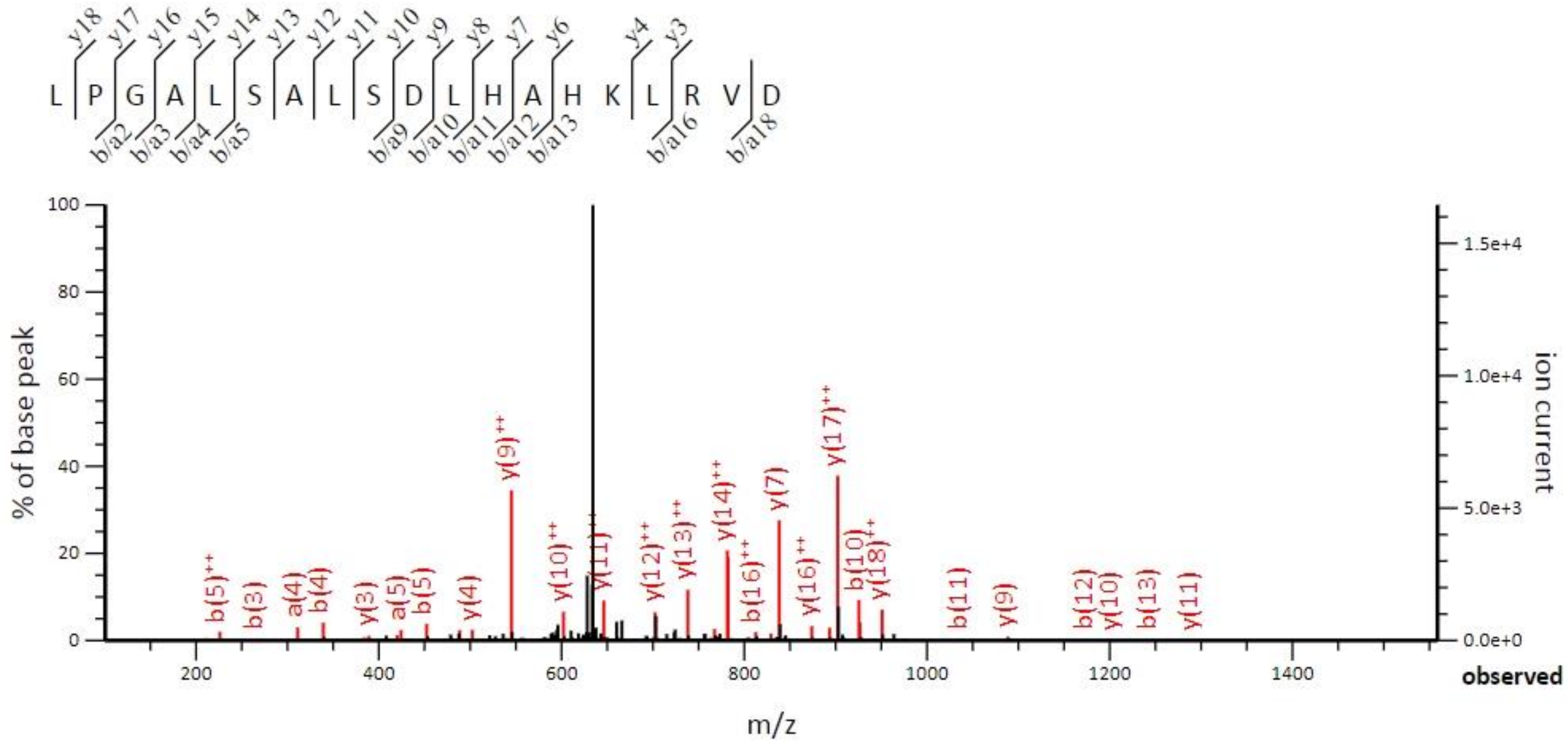

Figure S13: Deconvolution of the main peptide from the fraction A3 (eluted at 81.8 min) corresponding to the fragment 77-95 of hemoglobin α chain.

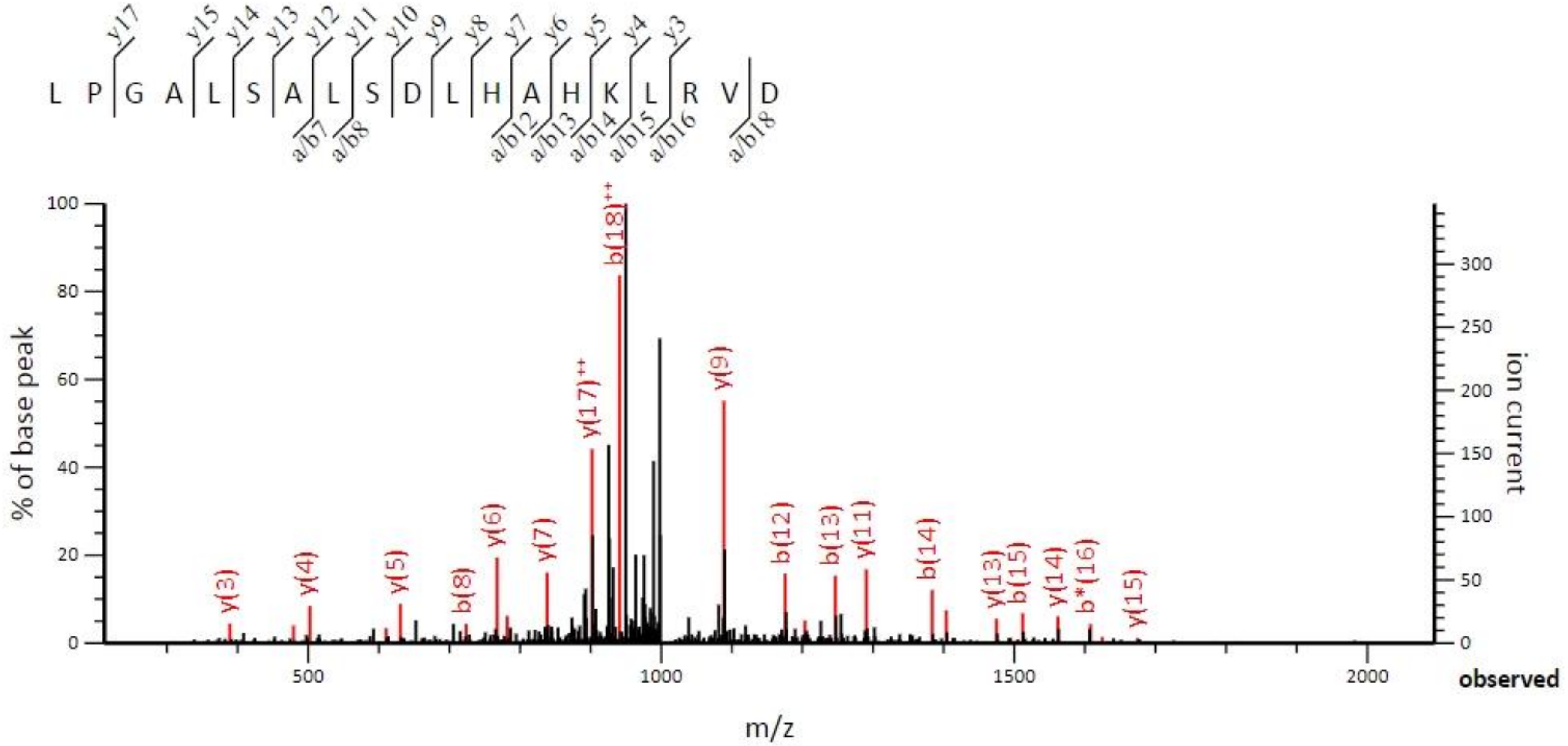

Figure S14: Deconvolution of the main peptide from the fraction B1 (eluted at 28.8 min) corresponding to the fragment 2-10 of hemoglobin β2 chain.

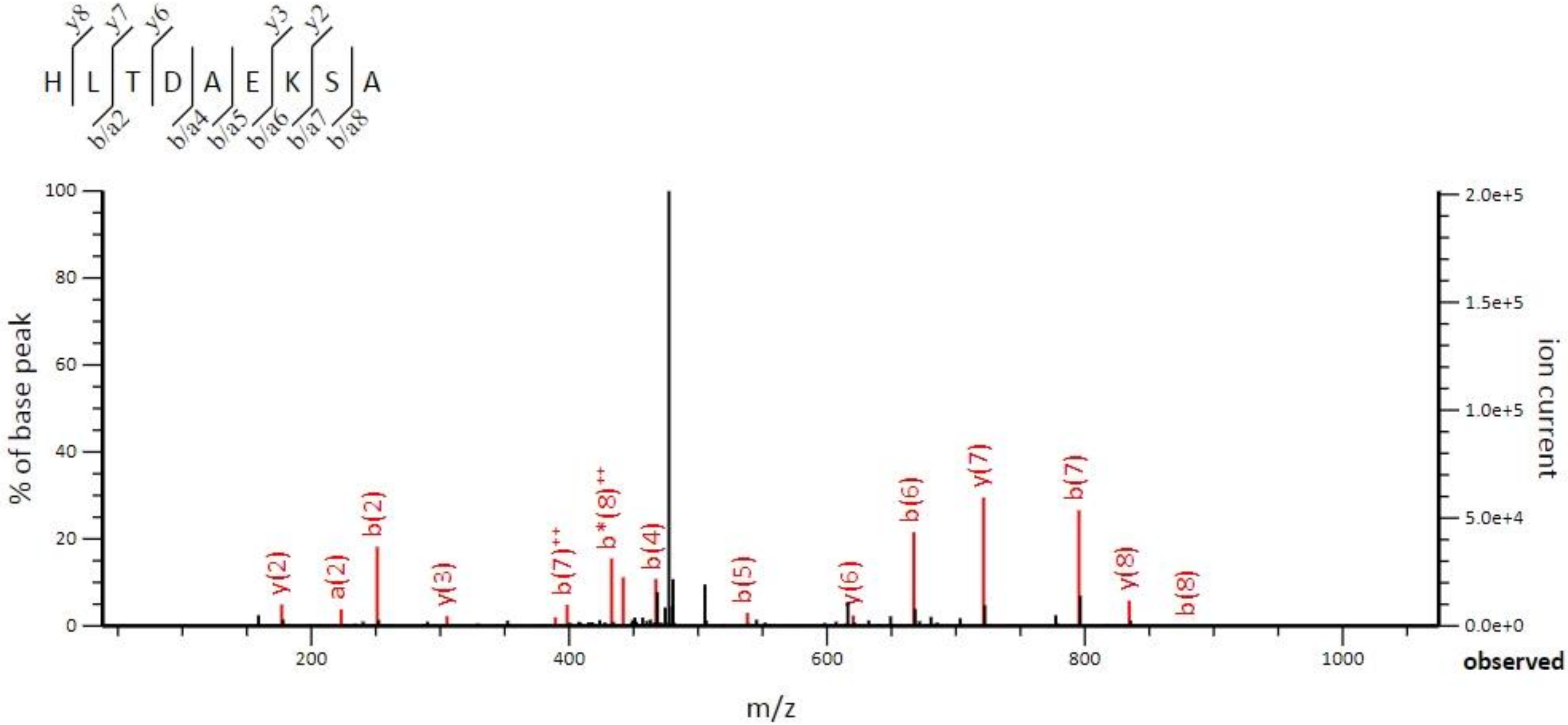

Figure S15: Deconvolution of the main peptide from the fraction B2 (eluted at 35.5 min) corresponding to the fragment 1-10 of hemoglobin β2 chain.

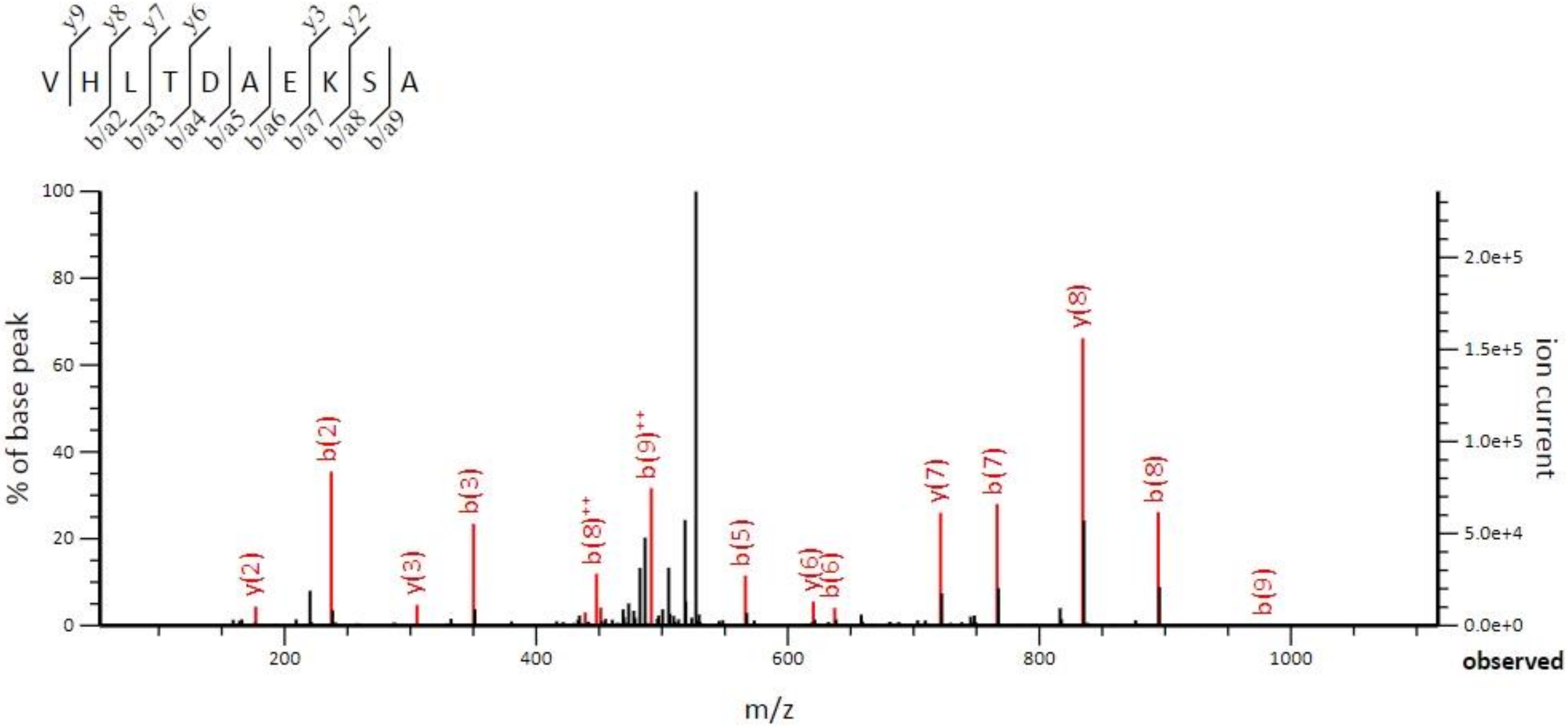

Figure S16: Deconvolution of the main peptide from the fraction B3 (eluted at 43.0 min) corresponding to the fragment 59-74 of hemoglobin β1 chain.

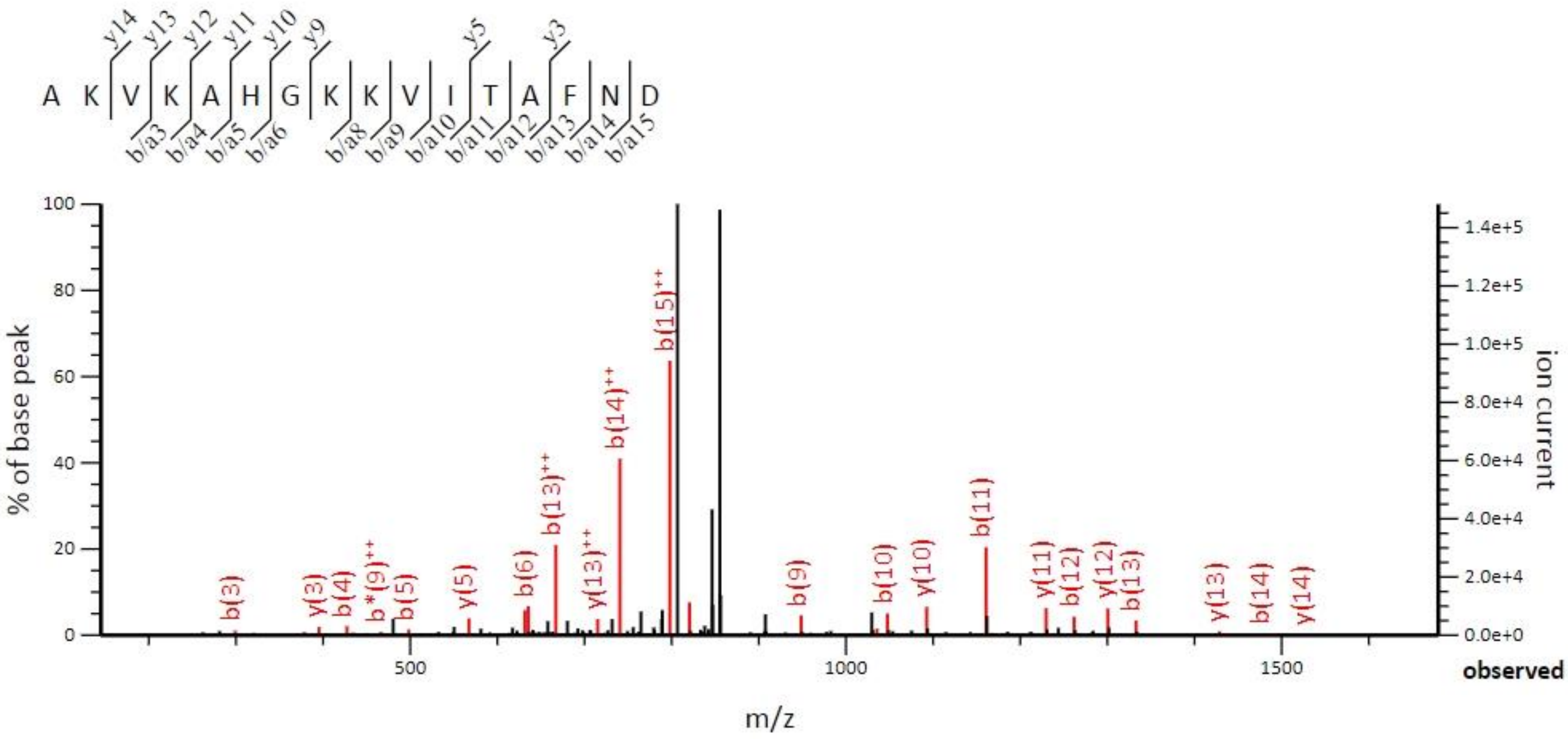

Figure S17: Deconvolution of the main peptide from the fraction B4 (eluted at 48.1 min) corresponding to the fragment 59-77 of hemoglobin β1 chain.

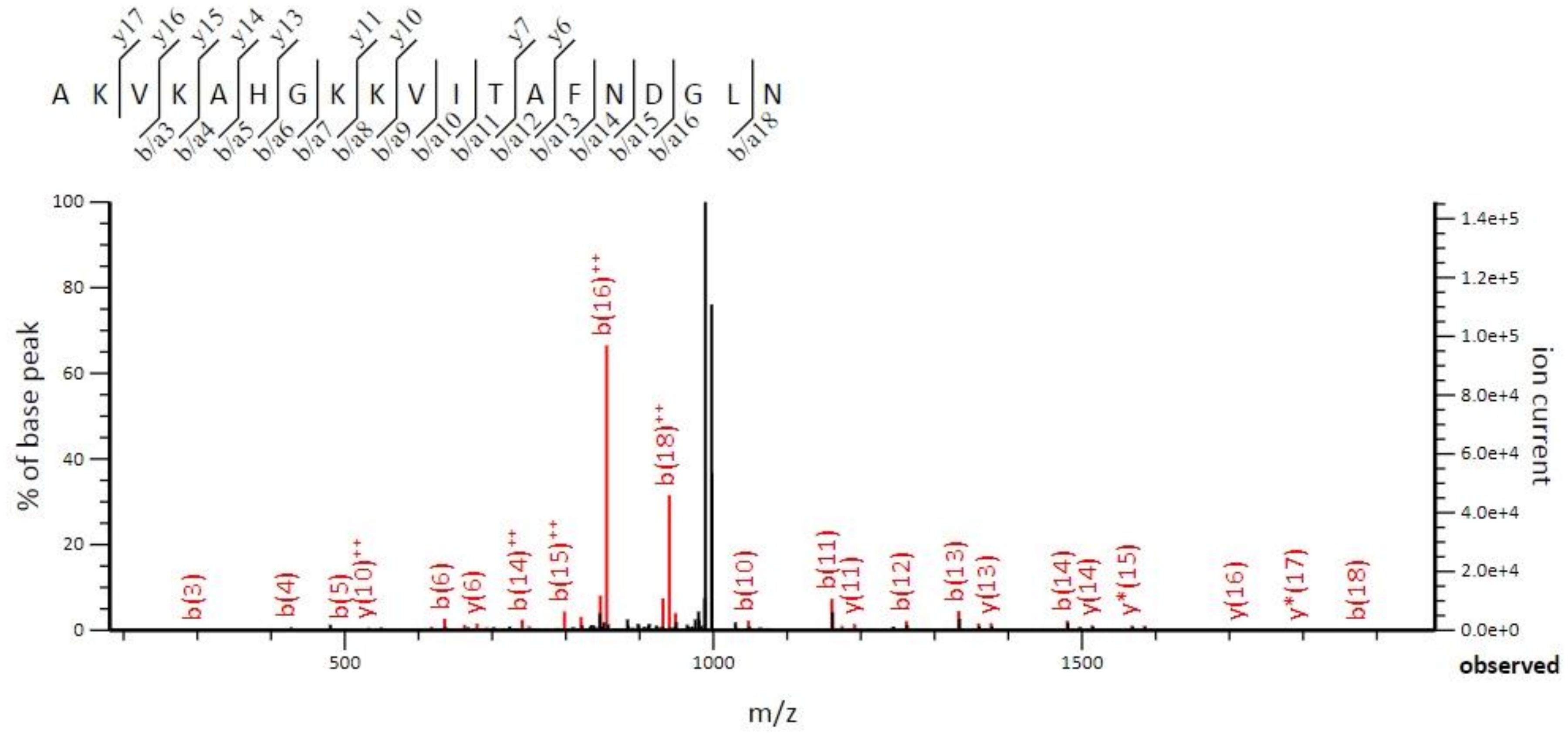

Figure S18: Deconvolution of the main peptide from the fraction B5 (eluted at 56.4 min) corresponding to the fragment 75-84 of hemoglobin β1 chain.

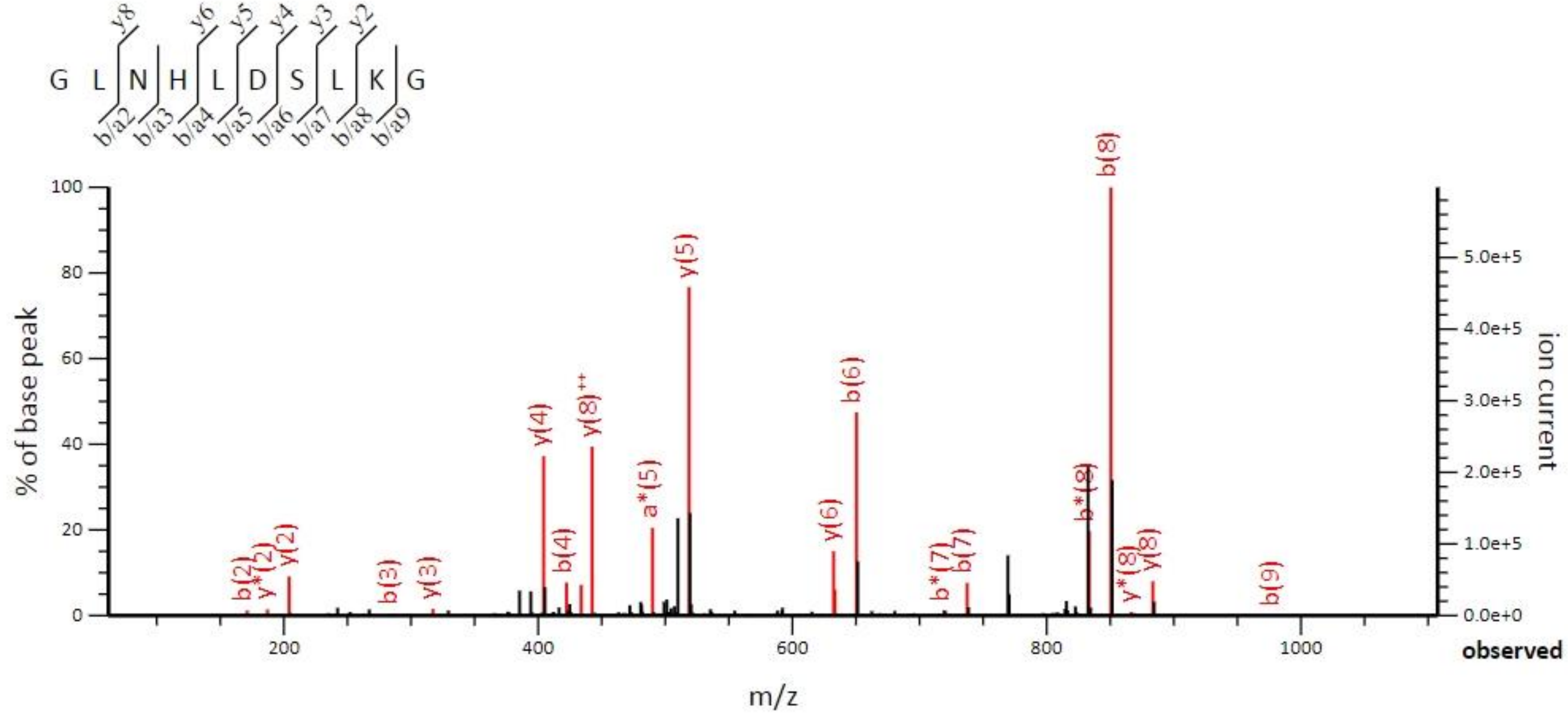

Figure S19: Deconvolution of the main peptide from the fraction B6 (eluted at 61.7 min) corresponding to the fragment 131-147 of hemoglobin β1 chain.

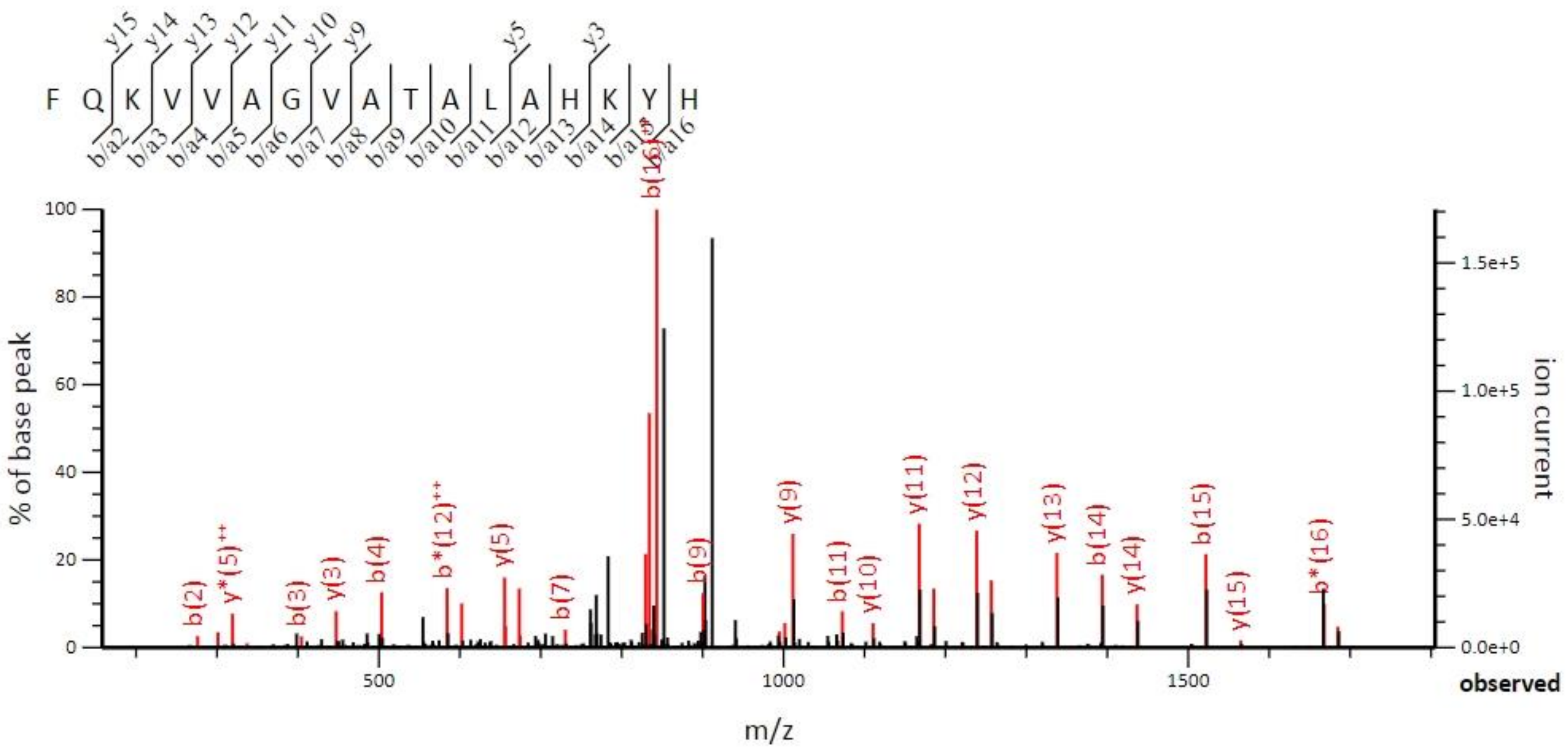

Figure S20: Deconvolution of the main peptide from the fraction B7 (eluted at 93.7 min) corresponding to the fragment 123-147 of hemoglobin β2 chain.

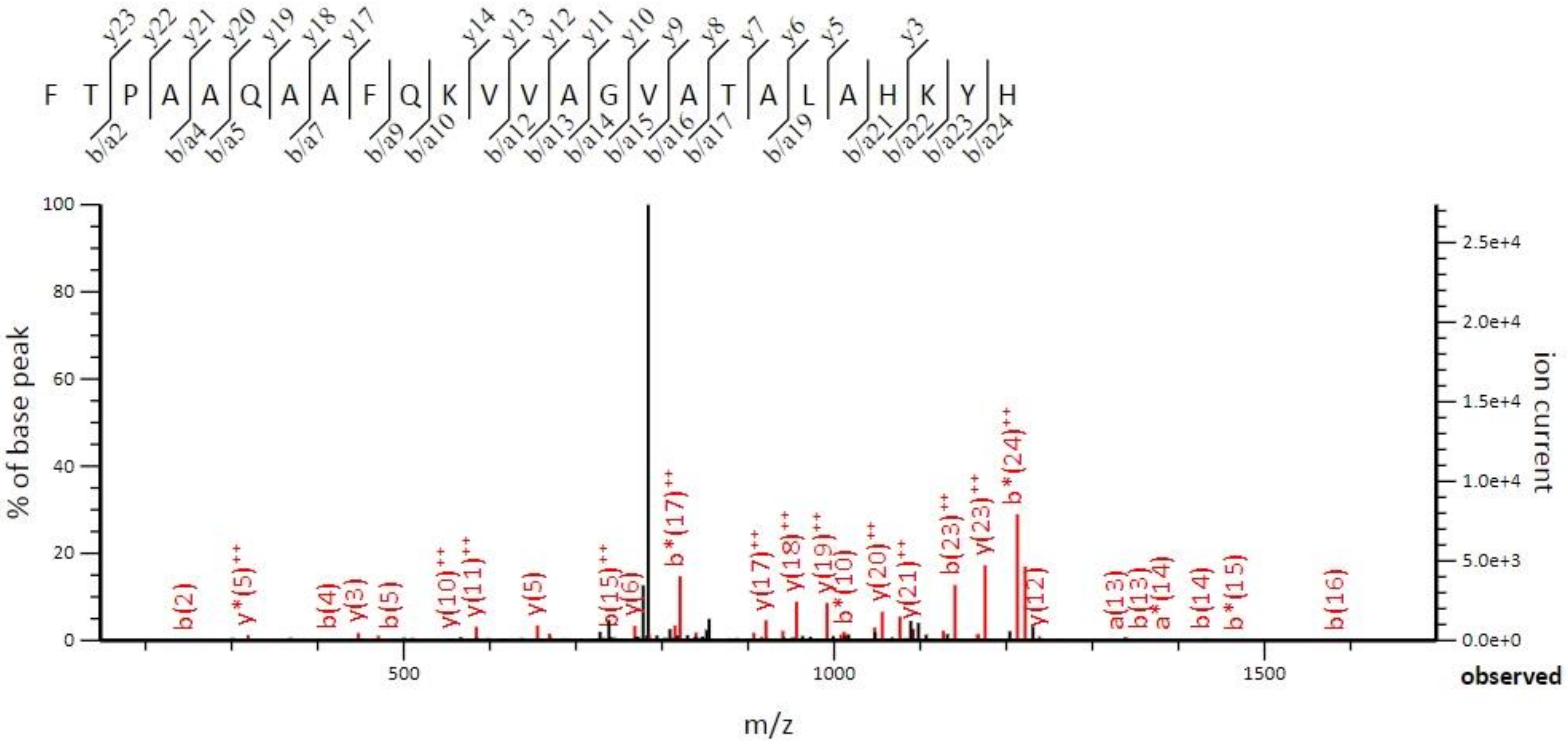

Supplement: Supplementary file 1 [file biomolecules-10-00261-s001.pdf]
